# Soft nanobrush-directed multifunctional MOF nanoarrays

**DOI:** 10.1038/s41467-022-34512-1

**Published:** 2022-11-05

**Authors:** Shuang Wang, Wenhe Xie, Ping Wu, Geyu Lin, Yan Cui, Jiawei Tao, Gaofeng Zeng, Yonghui Deng, Huibin Qiu

**Affiliations:** 1 School of Chemistry and Chemical Engineering, Zhangjiang Institute for Advanced Study, Frontiers Science Center for Transformative Molecules, State Key Laboratory of Metal Matrix Composites, Shanghai Jiao Tong University, Shanghai, 200240 P. R. China; 2 Department of Chemistry, State Key Laboratory of Molecular Engineering of Polymers, Shanghai Key Laboratory of Molecular Catalysis and Innovative Materials, iChEM, Fudan University, Shanghai, 200433 China; 3 CAS Key Laboratory of Low-carbon Conversion Science and Engineering, Shanghai Advanced Research Institute, Chinese Academy of Sciences, 100 Haike Road, Shanghai, 201210 China

**Keywords:** Metal-organic frameworks, Heterogeneous catalysis, Metal-organic frameworks

## Abstract

Controlled growth of well-oriented metal-organic framework nanoarrays on requisite surfaces is of prominent significance for a broad range of applications such as catalysis, sensing, optics and electronics. Herein, we develop a highly flexible soft nanobrush-directed synthesis approach for precise in situ fabrication of MOF nanoarrays on diverse substrates. The soft nanobrushes are constructed via surface-initiated living crystallization-driven self-assembly and their active poly(2-vinylpyridine) corona captures abundant metal cations through coordination interactions. This allows the rapid heterogeneous growth of MOF nanoparticles and the subsequent formation of MIL-100 (Fe), HKUST-1 and CUT-8 (Cu) nanoarrays with tailored heights of 220~1100 nm on silicon wafer, Ni foam and ceramic tube. Auxiliary functional components including metal oxygen clusters and precious metal nanoparticles can be readily incorporated to finely fabricate hybrid structures with synergistic features. Remarkably, the MIL-100 (Fe) nanoarrays doped with Keggin H_3_PMo_10_V_2_O_40_ dramatically boost formaldehyde selectivity up to 92.8% in catalytic oxidation of methanol. Moreover, the HKUST-1 nanoarrays decorated with Pt nanoparticles show exceptional sensitivity to H_2_S with a ppb-level detection limit.

## Abstract (web summary)

Tailored growth of MOFs is of great interest for a broad range of applications. Here authors present the use of soft nanobrushes to direct the growth of MOF nanoarrays on requisite substrates, which simultaneously allows the loading of auxiliary functional species for advanced catalysis and sensing applications.

## Introduction

**Fig. 1:**
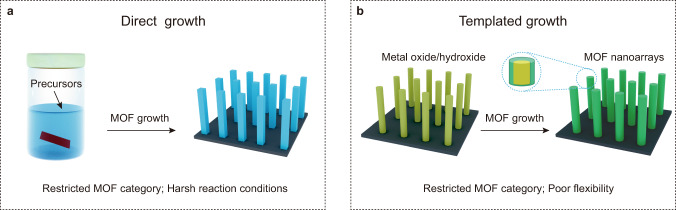
Traditional routes to MOF nanoarrays. **a** Direct growth of MOF nanoarrays under solvothermal conditions. **b** Directed growth of MOF nanoarrays on hard templates.

Nanoarrays that combine the characteristics of nanosize and directional arrangement have aroused widespread attention in a variety of fileds^1–5^. In particular, metal-organic frameworks (MOFs) featuring tailorable pore dimension and chemical functionality present prominent advantages in the construction of multifunctional nanoarrays^6–8^. The uniform alignment of intrinsically porous nanopillars creates a hierarchically open environment for full exposure of active sites and free transfer of reactive substrates, which is favorable for catalysis, sensor, lithium storage, and drug delivery^9–11^. Generally, highly oriented MOF nanoarrays may grow directly on substrates through “one-pot” solvothermal reactions (Fig. 1a). This approach is simple yet powerful, but usually requires selected MOFs with inherent one- or two-dimensional crystalline structures^12,13^. Alternatively, template-directed synthesis is applied to construct multidimensional MOF nanoarrays (Fig. 1b). Notably, metal oxides and hydroxides [e.g., ZnO, CuO, Cu(OH)_2_ and CoO] arrays have been widely used as hard templates to direct the growth of MOF nanoarrays^14–17^. While these templates facilitate the nucleation of MOFs and rationally direct the growth of MOFs along the templates^18^, they normally decompose to release essential metal sources for the growth of desired MOFs. Consequently, the category of MOF nanoarrays is substantially limited by the composition of templates. Besides, it remains a major challenge to handily manipulate the height of the nanoarrays or to simultaneously introduce complementary functional species.

Here, we develop a highly flexible soft nanobrush-directed strategy for the precise fabrication of MOF nanoarrays. The soft nanobrushes are facilely introduced onto various substrates via living crystallization-driven self-assembly^19^, and their abundant pyridine groups provide the active sites for the capture of copious metal cations to direct the growth of diverse MOFs. By simply immersing in specific precursor solutions, various well-aligned MOF nanoarrays with high aspect ratios and controllable heights can be readily grown on silicon wafer, Ni foam and ceramic tube. Subsequently, polyoxometalates and noble metal nanoparticles are elaborately introduced into the nanoarrays for synergistic catalytic oxidation of methanol and ultra-sensitive sensing of hydrogen sulfide.

## Results and discussion

**Fig. 2:**
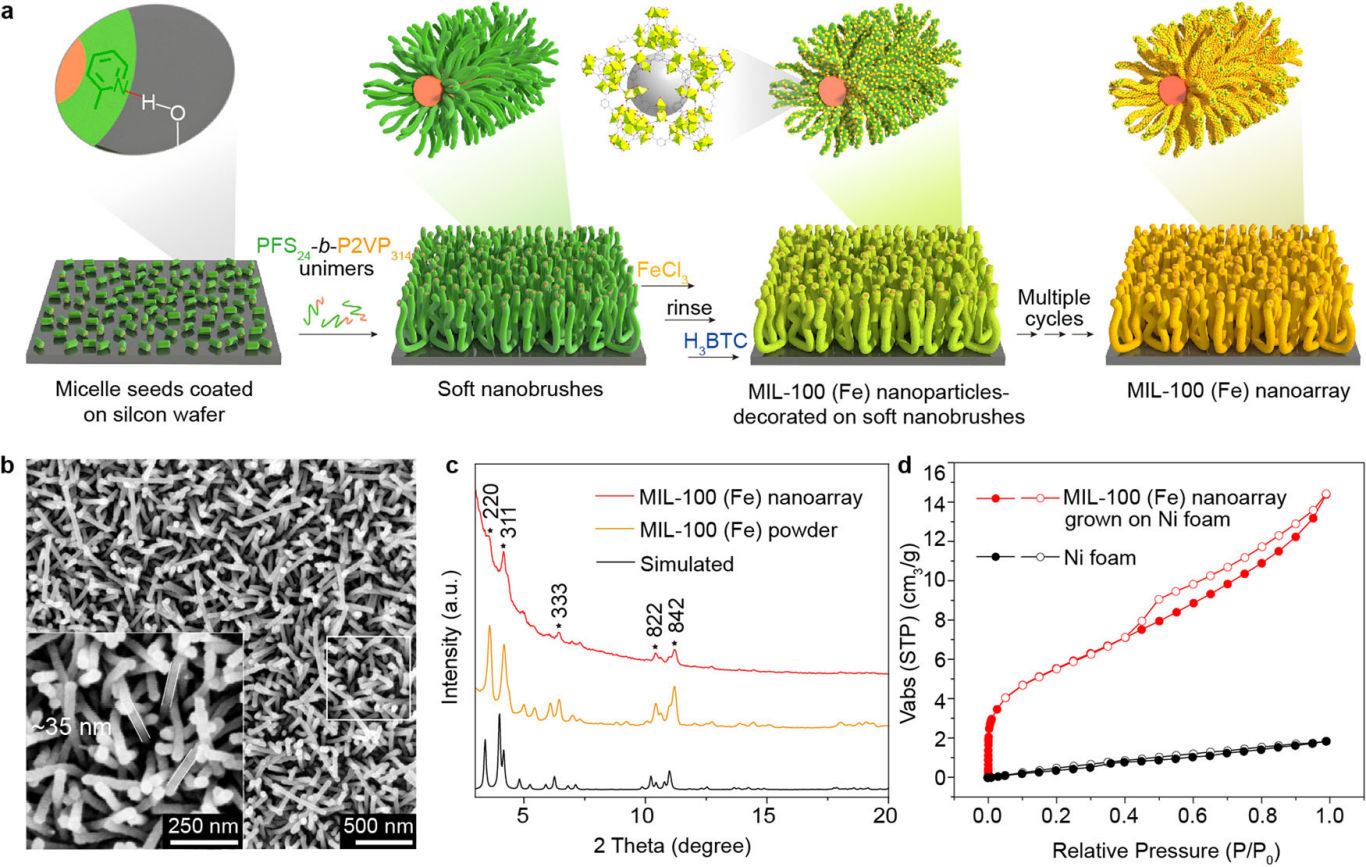
Soft nanobrush-directed growth of MIL-100 (Fe) nanoarrays. **a** Schematic illustration of the fabrication process. **b** Scanning electron microscope (SEM) images of a nanoarray obtained by alternately immersing a soft nanobrush-coated silicon wafer into ethanol solutions of FeCl_3_ and H_3_BTC for five cycles. **c** XRD pattern of the obtained MIL-100 (Fe) nanoarray with comparison to that of the MIL-100 (Fe) powder and simulated XRD pattern of MIL-100 (Fe). **d** N_2_ sorption isotherms of a piece of MIL-100 (Fe) nanoarray-decorated Ni foam and a piece of naked Ni foam. Source data are provided as a Source Data file.

Soft nanobrush was firstly prepared on a silicon wafer via surface-initiated living crystallization-driven self-assembly of PFS_24_-*b*-P2VP_314_ on pre-immobilized PFS_44_-*b*-P2VP_526_ seeds [PFS = polyferrocenyldimethylsilane, P2VP = poly(2-vinylpyridine), the subscripts refer to the number-average degree of polymerization of each block] (Supplementary Fig. 1)^19^. The abundant pyridine groups in the P2VP corona were capable to bind with various metal ions through coordination interactions^20^. Initially, FeCl_3_ and 1,3,5-benzenetricarboxylic acid (H_3_BTC) were introduced simultaneously into the soft nanobrush system, but it unfortunately led to the formation of irregular composites (Supplementary Fig. 2). Nevertheless, by alternately immersing the soft nanobrush-coated silicon wafer into ethanol solutions of FeCl_3_ and H_3_BTC at room temperature five times (Fig. 2a), a uniform array of nanorods with a high aspect ratio and an average diameter of ~35 nm was eventually obtained (Fig. 2b). The contour of each nanorod was obviously sharper compared to the soft nanobrush (Supplementary Fig. 3), indicating a fine coating of nanoparticles on the soft nanobrush. Upon further increasing the immersing cycles, the diameter of the nanorods increased to ~43 nm for eight cycles and to ~55 nm for ten cycles (Supplementary Fig. 4). In contrast, no growth of nanoarray was observed on naked silicon wafers (Supplementary Fig. 5). X-ray diffraction (XRD) pattern of the nanoarray revealed relatively strong peaks at 3.5°, 4.1°, 6.4°, 10.4° and 11.2° (Fig. 2c), which can be assigned to the (220), (311), (333), (822) and (842) planes of MIL-100 (Fe), respectively. X-ray photoelectron spectroscopy (XPS) spectra of N 1 *s* of the MIL-100 (Fe) nanoarray shifted to a higher binding energy value (399.68 eV) compared to the pristine soft nanobrush (398.96 eV) (Supplementary Fig. 6), indicating a strong coordination interaction between the pyridine groups on the soft nanobrush with the Fe centers in the MIL-100 (Fe) nanoparticles. N_2_ sorption isotherm of the MIL-100 (Fe) nanoarray grown on a piece of Ni foam (Supplementary Fig. 7) showed a prominent sorption in a low relative pressure region (Fig. 2d), revealing a typical microporous feature for MIL-100 (Fe).

**Fig. 3:**
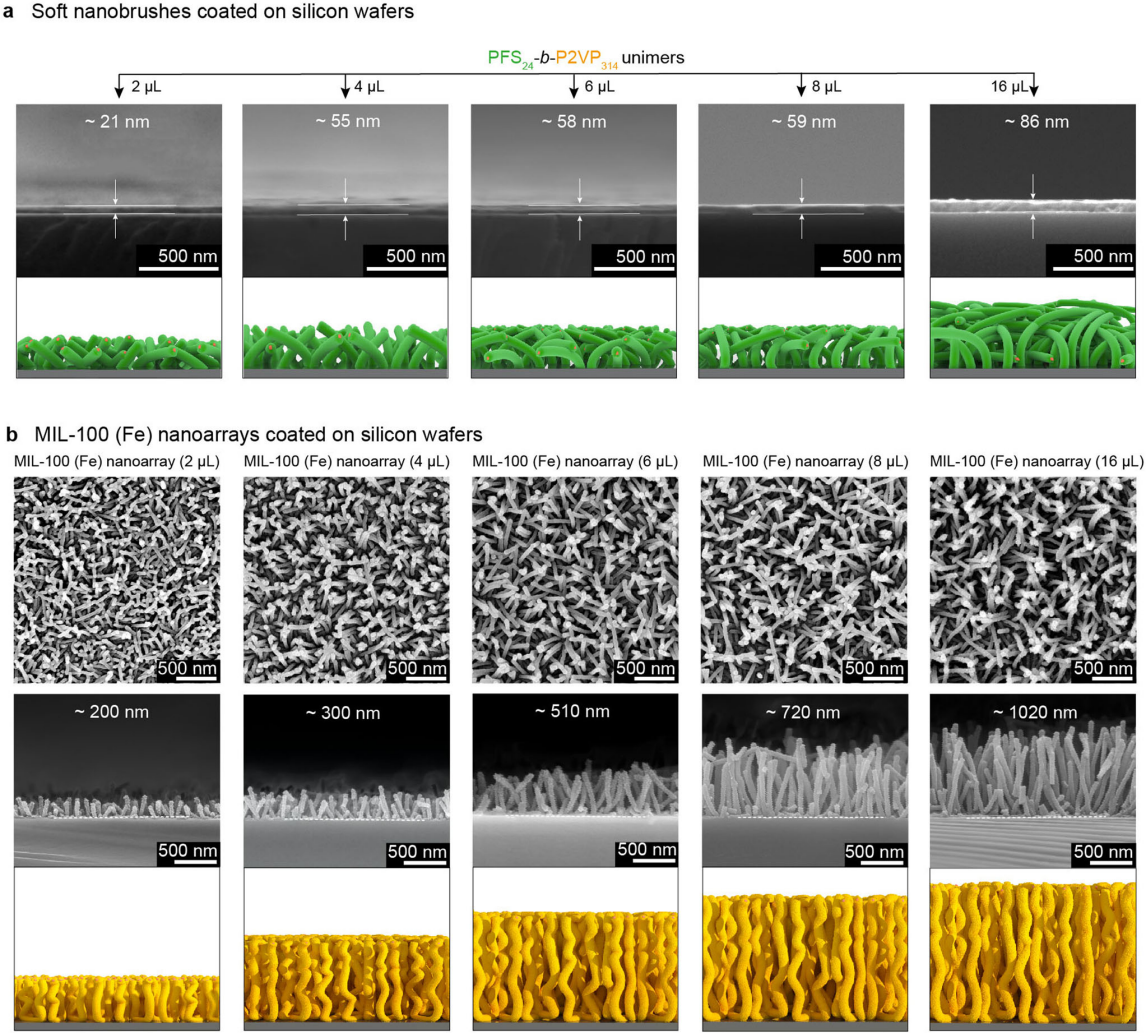
Height regulation of MIL-100 (Fe) nanoarrays. **a** Cross-sectional SEM images of soft nanobrushes formed with the addition of 2, 4, 6, 8 and 16 µL of a THF solution (10 mg/mL) of PFS_24_-*b*-P2VP_314_ unimers. **b** Top-view and cross-sectional SEM images of MIL-100 (Fe) nanoarrays directed by the corresponding soft nanobrushes shown in **a**.

Soft nanobrushes with variable lengths were further used to direct the growth of MIL-100 (Fe) nanoarrays. Cross-sectional SEM images showed that the height of the dried soft nanobrushes gradually increased from ~21 to ~86 nm with the addition of 2, 4, 6, 8 and 16 µL of a THF solution of PFS_24_-*b*-P2VP_314_ unimers (Fig. 3a, and Supplementary Fig. 8). In contrast to these mostly collapsed soft nanobrushes, the resultant MIL-100 (Fe) nanoarrays revealed erect morphologies, and their height was considerably higher and constantly increased from ~220 to ~1100 nm (Fig. 3b and Supplementary Fig. 9). Notably, the MIL-100 (Fe) nanoarrays were comprised of very uniform cylindrical pillars with a constant diameter of ~35 nm from root to top, indicating a highly controllable growth of MIL-100 (Fe) along the soft nanobrush. Generally, the height variation of the MIL-100 (Fe) nanoarrays was almost linearly consistent with the addition of PFS_24_-*b*-P2VP_314_ unimers (i.e. the length of the soft nanobrushes)^19^ (Supplementary Fig. 10). Consequently, it is very convenient to modulate the height of the MOF nanoarrays through surface-initiated living crystallization-driven self-assembly.

**Fig. 4:**
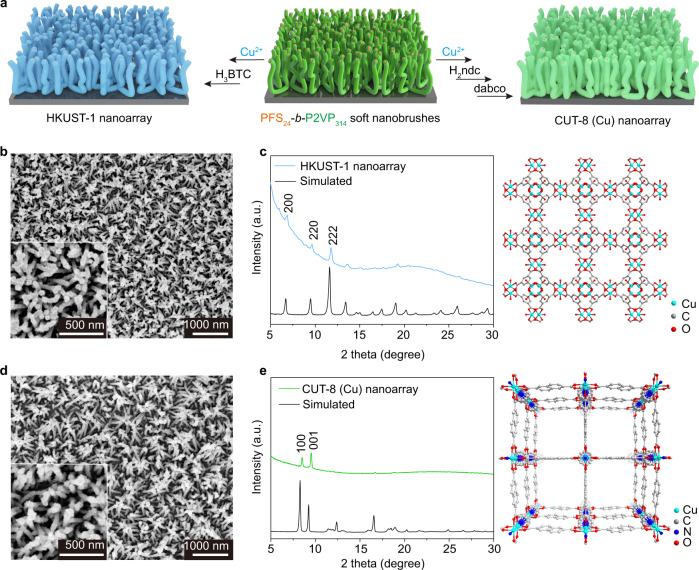
Extension of soft nanobrush-directed MOF nanoarrays. **a** Scheme illustration of the fabrication process for HKUST-1 and CUT-8 (Cu) nanoarrays. **b** SEM images of an HKUST-1 nanoarray obtained by alternately immersing a soft nanobrush-coated silicon wafer into ethanol solutions of Cu(CH_3_COO)_2_ and H_3_BTC for five cycles. **c** XRD pattern of the obtained HKUST-1 nanoarray and simulated XRD pattern of HKUST-1. The right image shows the crystal structure of HKUST-1. **d** SEM images of a CUT-8 (Cu) nanoarray obtained by alternately immersing a soft nanobrush-coated silicon wafer into ethanol solutions of Cu(CH_3_COO)_2_, H_2_ndc and dabco for five cycles. **e** XRD pattern of the obtained CUT-8 (Cu) nanoarray and simulated XRD pattern of CUT-8 (Cu). The right image shows the crystal structure of CUT-8 (Cu). The soft nanobrushes were formed by adding 6 μL of a solution of PFS_24_-*b*-P2VP_314_ unimers (10 mg/mL in THF). Source data are provided as a Source Data file.

To explore the synthesis generality, the soft nanobrushes were further employed to direct the growth of HKUST-1 nanoarrays by repeatedly immersing the soft nanobrush-coated silicon wafer into ethanol solutions of Cu(OAC)_2_ and H_3_BTC five times (Fig. 4a, see Supplementary Figs. S11–14 for more details). The resultant nanoarrays were also comprised of well-defined rod-like pillars with an average diameter of ~45 nm (Fig. 4b). XRD pattern of the nanoarray revealed characteristic peaks for HKUST-1 at 6.8° (200), 9.5° (220) and 11.7° (222), respectively (Fig. 4c). High-angle annular dark-field scanning transmission electron microscopy (HAADF-STEM) confirmed the formation of hard rods with a clear profile, and the corresponding element mapping images demonstrated the typical composition of HKUST-1 (Supplementary Fig. 11f). Furthermore, CUT-8 (Cu) nanoarrays with dual-ligands were also fabricated on silicon wafers by repeatedly immersing in ethanol solutions of Cu(OAC)_2_, H_2_ndc (1,4-naphthalene-bdc) and dabco (1,4-diazabicyclo[2.2.2]octane) (Fig. 4d,e and Supplementary Fig. 15). Hence, the soft nanobrushes are highly promising to direct the growth of diverse MOF nanoarrays, aiming at various functions and applications.

**Fig. 5:**
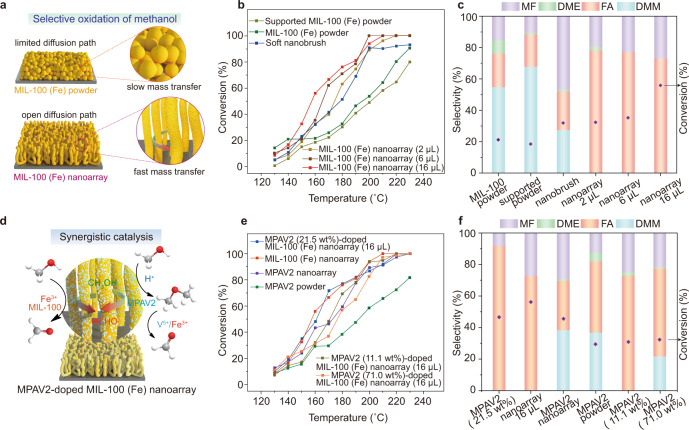
Selective oxidation of methanol catalyzed by MIL-100 (Fe) nanoarrays. **a** Schematic illustration of the catalytic environment of a MIL-100 (Fe) nanoarray and a layer of MIL-100 (Fe) powder. **b**, **c** Temperature dependence of conversion (**b**) and FA selectivity (**c**) at 160 °C for the oxidation of methanol in the presence of the pristine MIL-100 (Fe) powder, MIL-100 (Fe) powder immobilized on a silicon wafer, soft nanobrushes and MIL-100 (Fe) nanoarrays (2 µL, 6 µL, 16 µL), respectively. **d** Schematic illustration of the synergistic catalytic effect between the MIL-100 (Fe) nanoarray and MPAV2 during the oxidation of methanol. **e**, **f** Temperature dependence of conversion (**e**) and FA selectivity (**f**) at 160 °C for the oxidation of methanol in the presence of the MPAV2 (21.5 wt%)-doped MIL-100 (Fe) nanoarray (16 µL), MIL-100 (Fe) nanoarray (16 µL), MPAV2 nanoarray, MPAV2 powder, MPAV2 (11.1 wt%)-doped MIL-100 (Fe) nanoarray (16 µL) and MPAV2 (71.0 wt%)-doped MIL-100 (Fe) nanoarray (16 µL), respectively. Note: DMM = dimethoxymethane, DME = dimethyl ether. Source data are provided as a Source Data file.

MOF nanoarrays provided a remarkable platform for catalysis in consideration of the open and free space as well as the abundant metallic active sites^9^. It was previously found that Fe-based materials are promising redox catalysts for selective oxidation of CH_3_OH into formaldehyde (FA), an important chemical intermediate for polyacetal resin and adhesive, via oxidative dehydrogenation^21–23^. Consequently, the Fe-containing MIL-100 nanoarrays were used to catalyze the oxidation of methanol under industrially relevant conditions (Fig. 5a). Compared to the pristine or supported (immobilized on a silicon wafer) MIL-100 (Fe) powder, the MIL-100 (Fe) nanoarrays (2 µL, 6 µL, 16 µL) showed remarkably higher activity at higher temperatures (>160 °C) and enabled 100% conversion of methanol over 200 °C (Fig. 5b). As a reflection of the intrinsic activity of the iron center, the turnover frequency (TOF) of the MIL-100 (Fe) nanoarray (16 µL) (216.25 h^−1^) was also obviously higher than that of the pristine (0.0355 h^−1^) and supported MIL-100 (Fe) powder (0.773 h^−1^) (Supplementary Table 1), Besides, the erect MIL-100 (Fe) nanoarray showed a significantly faster reaction rate than the collapsed sample (Supplementary Fig. 16), indicating a faster mass transfer within the highly open MIL-100 (Fe) nanoarray. Besides, the MIL-100 (Fe) nanoarray (16 µL) exhibited a remarkably higher maximum (at 160 °C, Supplementary Fig. 17) FA selectivity of 72.7% compared to the pristine (21.4%) and supported MIL-100 (Fe) powder (20.6%), as well as the soft nanobrush (<20%) (Fig. 5c). It should be noted that the morphology of the MIL-100 (Fe) nanoarrays was well preserved after the catalytic reaction (Supplementary Fig. 18), indicative of superior structural stability.

Previous studies have demonstrated that the addition of vanadium centers can considerably boost the selectivity of FA via promoting the dehydrogenation of CH_3_OH molecules^24,25^. However, these vanadium-containing active components were rarely incorporated into MOF arrays probably due to the unfavorable interactions. Fortunately, the P2VP corona of the soft nanobrush can simultaneously bind with the vanadium species in the growth process of MOFs and hence may function as a Trojan horse to incorporate the vanadium-based components into the MOF nanoarrays. To this end, the soft nanobrush-coated silicon wafer was additionally immersed in an ethanol solution of vanadium-substituted Keggin polyoxometalate (H_3_PMo_10_V_2_O_40_, MPAV2) during the growth of the MIL-100 (Fe) nanoarray (Fig. 5d and Supplementary Fig. 19)^26^. Element mapping of the hybrid nanoarray revealed a uniform distribution of MPVA2 throughout the MIL-100 (Fe) nanorods (Supplementary Fig. 19c). The hybrid nanoarray doped with 21.5 wt% of MPAV2 exhibited an initial reaction rate of 0.0198 mmol·g^−1^·min^−1^, which was significantly higher than that of the MIL-100 (Fe) nanoarrays doped with 11.1 and 71.0 wt% of MPAV2, and the collapsed MIL-100 (Fe) doped with 21.5 wt% of MPAV2 (Fig. 5e and Supplementary Fig. 20). Meanwhile, the nanoarray doped with 21.5 wt% of MPAV2 revealed a significantly enhanced FA selectivity of 92.8%, much higher than the MIL-100 (Fe) nanoarray (16 µL) (72.7%), the MPAV2 powder (42.7%), the pristine MPAV2 nanoarray (16 µL) and the previously reported Fe-based materials (Fig. 5f and Supplementary Table 2). These findings demonstrated the presence of a synergistic catalytic effect between the MPVA2 and MIL-100 (Fe), where the abundant Fe^3+^ active sites on the highly aligned MIL-100 (Fe) nanoarray dominate the oxidative dehydrogenation of CH_3_OH, while the doped MPAV2 moieties provide additional redox catalytic sites (V^5+^) and acid sites involved in the acetalization reactions. On the contrary, the phosphotungstic acid PW_12_ only provided the acid sites^27^ and hence the PW_12_ (18.6 wt%)-doped MIL-100 (Fe) nanoarray only led to the formation of MF (methyl formate) as the major product (Supplementary Fig. 21). Notably, the morphology and catalytic activity of the nanoarray doped with 21.5 wt% of MPAV2 were well retained after 7 cycles of methanol oxidation (Supplementary Figs. 22, 23), indicative of a high recyclability.

**Fig. 6:**
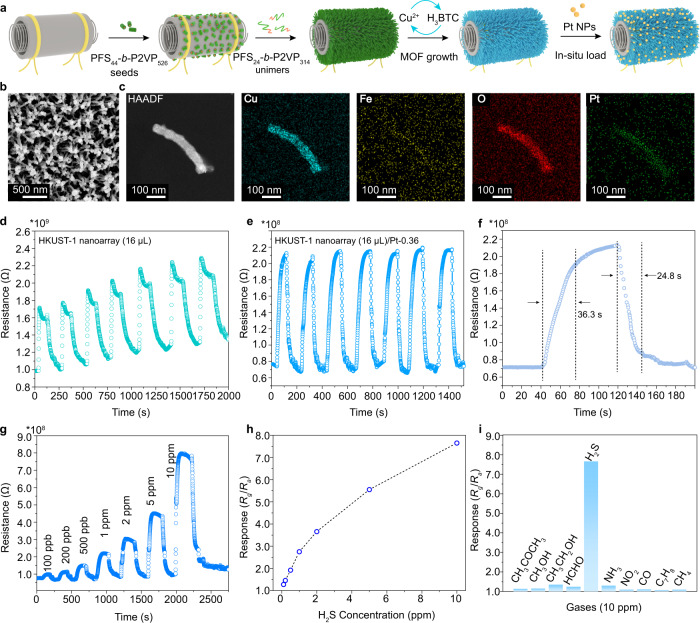
Fabrication of HKUST-1 nanoarrays on ceramic tubes and their gas sensing performance. **a** Schematic illustration of the fabrication process of HKUST-1 nanoarray on a ceramic tube and the further loading of Pt nanoparticles. **b** SEM image of the HKUST-1 nanoarray (16 µL)/Pt-0.36. **c** HRTEM image and element mapping of the HKUST-1 nanoarray (16 µL)/Pt-0.36. **d** Repeating response-recovery curve of the pristine HKUST-1 nanoarray (16 µL) to 1 ppm of H_2_S at 200 °C. **e** Repeating response-recovery curve of the HKUST-1 nanoarray (16 µL)/Pt-0.36 to 1 ppm of H_2_S at 200 °C. **f** Fine analysis of a response-recovery cycle of the HKUST-1 nanoarray (16 µL)/Pt-0.36 to 1 ppm H_2_S at 200 °C. **g** Response-recovery curve of the HKUST-1 nanoarray (16 µL)/Pt-0.36 to H_2_S of different concentrations at 200 °C. **h** Response (*S* = *R*_*g*_/*R*_*a*_) of the HKUST-1 nanoarray (16 µL)/Pt-0.36 versus H_2_S concentration. **i** Responses of the HKUST-1 nanoarray (16 µL)/Pt-0.36 to different chemical vapors (10 ppm) at 200 °C. Source data are provided as a Source Data file.

The MOF nanoarrays also provided a promising platform for gas sensing because of the highly open structure and abundant metal active sites^28^. To this end, the HKUST-1 nanoarrays were specifically grown on the ceramic tubes devices (Fig. 6a and Supplementary Figs. 24, 25) with an aim to study their gas sensing performance toward H_2_S by taking advantage of the affinity of cupric ions for H_2_S^29,30^. Unfortunately, the pristine HKUST-1 nanoarrays only showed irreversible response to H_2_S (1 ppm) even at 200 °C, probably as a consequence of its poor conductivity and strong binding with H_2_S (Fig. 6d). To solve this problem, Pt nanoparticles were deliberately introduced to the HKUST-1 nanoarray through in situ reduction to improve the conductivity and increase the desorption rate of H_2_S (Fig. 6b,c and Supplementary Figs. 26–31)^31^. Compared to the pristine HKUST-1 nanoarray (16 µL), the HKUST-1 nanoarray (16 µL) loaded with 0.36 wt% of Pt nanoparticles [HKUST-1 nanoarray (16 µL)/Pt-0.36] displayed a remarkably higher sensitivity and cycling reversibility to 1 ppm H_2_S at 200 °C and the resistance quickly recovered to the baseline within 17.9 s (Fig. 6e and Supplementary Figs. 32, 33). Meanwhile, the resistance value of the HKUST-1 nanoarray (16 µL)/Pt-0.36 reached 2.1 × 10^8^ Ω, which was a magnitude lower than the pristine HKUST-1 nanoarray (16 µL) (2.3 × 10^9^ Ω). As a parameter to evaluate the desorption dynamics, the recovery time (24.8 s) of the HKUST-1 nanoarray (16 µL)/Pt-0.36 in 1 ppm H_2_S was apparently shorter than that of the pristine HKUST-1 nanoarrays (over 125 s) and the collapsed HKUST-1 nanoarray (16 µL)/Pt-0.36 (37 s) (Fig. 6f, and Supplementary Figs. 34, 35), indicating a fast desorption of H_2_S. Besides, the HKUST-1 nanoarray (16 µL)/Pt-0.36 showed a quick response to H_2_S with various concentrations from 0.1 to 10 ppm (Fig. 6g) and the response value (*S* = *R*_*g*_/*R*_*a*_) continuously increased from 1.22 to 7.55 (Fig. 6h). XPS analysis and density functional theory (DFT) calculations both showed that the transformation of H_2_S molecules is dynamically and thermodynamically more favorable on the HKUST-1/Pt nanoarray (Supplementary Figs. 36–38). Notably, the HKUST-1 nanoarray (16 µL)/Pt-0.36 achieved an ultrahigh selectivity toward H_2_S at 10 ppm with negligible response to the other interference chemical vapors (Fig. 6i). Moreover, the sensing performance of the HKUST-1 nanoarray (16 µL)/Pt-0.36 remained at a relatively high level over 2 weeks (Supplementary Fig. 39). Compared to the conventional sensing materials, the HKUST-1 nanoarray (16 µL)/Pt-0.36 displayed a prominent comprehensive sensing performance (Supplementary Table 3).

In summary, a soft nanobrush-directed growth strategy was developed for precise fabrication of MIL-100 (Fe), HKUST-1 and CUT-8 (Cu) nanoarrays on diverse substrates. The height of MOF nanoarrays was readily tailored from ~220 nm to ~1100 nm by precisely controlling the length of soft nanobrushes. The soft nanobrushes also provided an extremely flexible platform for efficient incorporation of a variety of complementary functional species, aiming at diverse synergistic features. Notably, additional doping of Keggin-type MPAV2 into the MIL-100 (Fe) nanoarray further improved the selectivity of FA to a remarkable value of 92.8% for the oxidation of methanol. Besides, the HKUST-1 nanoarrays loaded with 0.36 wt% of Pt nanoparticles presented exceptional sensitivity to H_2_S along with prominent cycling stability. It is expected that the soft nanobrush template approach demonstrated in this work would not only facilitate the growth of more types of MOFs but also offer a facile pathway to other inorganic functional nanoarrays.

## Methods

### Preparation of MIL-100 (Fe) nanoarray on silicon wafer

Typically, a silicon wafer coated with the PFS-*b*-P2VP soft nanobrush was immersed alternately in an ethanolic solution of FeCl_3_·6H_2_O (10 mM, 1 mL) for 10 min and then in an ethanolic solution of 1,3,5-benzene tricarboxylic acid (H_3_BTC) (10 mM, 1 mL) for 10 min at room temperature in a static reaction vessel. Between each step, the sample was rinsed with 1 mL of ethanol to remove the excess reagent. This process was repeated 5 times and the resulting sample was then lyophilized to obtain the MIL-100 (Fe) nanoarray.

### Preparation of MIL-100 (Fe) nanoarray on Ni foam

Typically, a piece of Ni foam coated with the PFS-*b*-P2VP soft nanobrush was immersed alternately in an ethanolic solution of FeCl_3_·6H_2_O (10 mM, 1 mL) for 10 min and then in an ethanolic solution of 1,3,5-benzene tricarboxylic acid (H_3_BTC) (10 mM, 1 mL) for 10 min at room temperature in a static reaction vessel. Between each step, the sample was rinsed with 1 mL of ethanol to remove the excess reagent. This process was repeated 5 times and the resulting sample was then lyophilized to obtain the MIL-100 (Fe) nanoarray.

### Preparation of HKUST-1 nanoarray on silicon wafer

Typically, a silicon wafer coated with the PFS-*b*-P2VP soft nanobrush was dipped alternately in an ethanolic solution of (Cu(CH_3_COO)_2_·H_2_O (10 mM, 1 mL)) for 10 min and then in an ethanolic solution of 1,3,5-benzene tricarboxylic acid (H_3_BTC) (10 mM, 1 mL) for 10 min at room temperature in a static reaction vessel. Between each step, the sample was rinsed with 1 mL of ethanol to remove the excess reagent. This process was repeated 5 times and the resulting sample was then lyophilized to obtain the HKUST-1 nanoarray. The HKUST-1 nanoarray was grown on a ceramic tube via a similar procedure.

### Preparation of CUT-8 (Cu) nanoarray on silicon wafer

Typically, a silicon wafer coated with the PFS-*b*-P2VP soft nanobrush was dipped alternately in an ethanolic solution of (Cu(CH_3_COO)_2_·H_2_O (2 mM, 1 mL)) for 10 min, an ethanolic solution of H_2_ndc (0.2 mM, 1 mL) for 10 min and an ethanolic solution of dabco (0.2 mM, 1 mL) for 10 min at room temperature in a static reaction vessel. Between each step, the sample was rinsed with 1 mL of ethanol to remove excess reagent. This process was repeated 5 times and the resulting sample was then lyophilized to obtain the CUT-8 (Cu) nanoarray.

### Preparation of MPAV2-doped MIL-100 nanoarray on silicon wafer

Typically, a silicon wafer coated with the PFS-*b*-P2VP soft nanobrush was dipped alternately in a mixed solution of water and ethanol (1:1 (v:v)) of MPAV2 (1 mM, 1 mL), an ethanolic solution of FeCl_3_·6H_2_O (10 mM, 1 mL) for 10 min and an ethanolic solution of 1,3,5-benzene tricarboxylic acid (H_3_BTC) (10 mM, 1 mL) for 10 min at room temperature in a static reaction vessel. Between each step, the sample was rinsed with 1 mL of ethanol to remove the excess reagent. This process was repeated 5 times and the resulting sample was then lyophilized to obtain the MPAV2-doped MIL-100 (Fe) nanoarray. PW_12_-doped MIL-100 (Fe) nanoarray was also fabricated via a similar procedure.

### Preparation of Pt-loaded HKUST-1 nanoarrays on ceramic tube

In a typical process, the ceramic tube coated with the HKUST-1 nanoarray was firstly placed in 0.5 mL of isopropanol and then 1 µL of an aqueous solution of Na_2_PtCl_4_·6H_2_O (1 mM) was added, and the mixture was shacked for 2 h. Subsequently, a chilled aqueous solution of NaBH_4_ (37.5 µL) was quickly added and the mixture was shaken on an oscillator for 20 min to allow the in situ formation of Pt nanoparticles. The resulting sample was rinsed with isopropanol and further lyophilized to obtain the Pt-loaded HKUST-1 nanoarray.

### Catalytic oxidation of methanol

The oxidation of methanol was performed in a fixed-bed tubular reactor with an inner diameter of 12 mm and a length of 550 mm. The catalyst sample was mixed with quartz sand (1.0 g) and then loaded into the reactor. Methanol was introduced into the reactor with a liquid hourly space velocity (LHSV) of 0.19 h^−1^ by a constant flow pump along with a flow of O_2_ (22.5 mL/min) and the gas hourly space velocity (GHSV) of reactant gases (methanol and oxygen) was 2832 mL/h/g. The products were analyzed by an on-line GC equipped with a thermal conductivity detector (TCD) and a Porapake-T column. The reaction was performed at atmospheric pressure and the gas lines between the reactor and GC were kept at 120 °C. The product selectivity was calculated as *S*_i_ (%) = n_i_/∑n_i_ × 100%, where i represents the specific product (CH_3_OCH_2_OCH_3_, HCOOCH_3_, HCHO, CH_3_OCH_3_, CO, or CO_2_), and n_i_ is the carbon atom molar of the specific product i.

### Gas sensing

The gas sensing tests were operated on a MA1.0 gas sensing measuring system (Narui Corp.Ltd., China). An alumina tube with a pair of printed Au electrodes printed on the black was used to support the sensing materials. A Ni-Cr alloy wire was equipped as a heater to provide tunable working temperature for sensing materials by applying a certain voltage. The assembled sensing device was then seated onto a PCB support with a pair of electrodes attached to the materials and allowed to age at 150 °C for 2 days to remove the excess solvent molecules in the MOF nanoarray. Target analyte, such as hydrogen sulfide, was passed into the gas sensor. The gas response of the sensor in this study was defined as *S* = *R*_g_/*R*_a_, where *R*_a_ is the sensor resistance in air and *R*_g_ is that in the gas tested. The response time was defined as the time required for the variation in conductance to reach 90% of the equilibrium value after a test gas was injected, and the recovery time was the time required for the sensor to return to 10% above the original conductance in air after releasing the test gas, respectively.

## Supplementary information

Supplementary Information

## Data Availability

Source data are provided with this paper.

## Source data

Source Data
