# Supplementary material for: Soft nanobrush-directed multifunctional MOF nanoarrays
Source: Nat Commun. 2022 Nov 5;13:6673. doi: 10.1038/s41467-022-34512-1 (PMC9637124; doi:10.1038/s41467-022-34512-1)
Supplement: Supplementary file 1 — Supplementary Information [file 41467_2022_34512_MOESM1_ESM.docx]

***Supplementary Information***

***Soft nanobrush-directed multifunctional MOF nanoarrays***

Wang et al.

**This PDF file includes:**

Supplementary Methods 2-4

Supplementary Figures 5-51

Supplementary References 52-54

**Supplementary Methods**

**Materials**

Copper (II) acetate monohydrate (Cu(CH_3_COO)_2_·H_2_O, 98%) and 1,3,5-benzenetricarboxylic acid (H_3_BTC, 99%) were purchased from Adamas-beta. Iron (III) chloride hexahydrate (FeCl_3_·6H_2_O, 99%) was purchased from Macklin. 1,4-Naphthalenedicarboxylic acid (H_2_ndc, 98%) and 1,4-Diazabicycle [2.2.2] octane (dabco, 99%) were obtained from Sinopharm Group Chemical Reagent Co., Ltd. All raw chemical reagents were used without any further purification. H_5_[PMo_10_V_2_O_40_]·32.5H_2_O (MPAV2) was synthesized according to the previously reported procedure^1^. PFS-*b*-P2VP block copolymers were synthesized via living anionic ROP as previously reported^2,3^.

**Characterization**

Atomic force microscopy (AFM) analysis was performed at ambient conditions using a Bruker Dimension FastScan atomic force microscope equipped with an Icon scanning module utilizing peak force feedback control. Samples in the dry state were characterized using a ScanAsyst-Air probe (tip radius, 2 nm) under the mode of ScanAsyst in air. Images were analyzed by NanoScope Analysis (version 1.80, an open-source software program developed for AFM images). Scanning electron microscopy (SEM) images were obtained on a TESCAN MAIA3 microscope or a JEOL JSM7800F Prime microscope both operating at 5.0 kV. Before SEM characterization, the samples were sputter-coated with a thin layer of platinum. Transmission electron microscopy (TEM) micrographs were obtained on a Talos L120C G2 microscope operating at 120 kV or a TALOS F200X microscope operating at 200 kV. High-resolution TEM (HR-TEM) and high-angle annular dark-field STEM (HAADF-STEM) micrographs and the corresponding elemental mapping images were obtained on a Thermo Scientific Talos F200X microscope or a Tecnai G2 F20S-Twin field emission TEM (FEI, America) both operating at 200 kV. Samples were prepared by placing one drop of solution on a carbon film-coated molybdenum grid, touching the edge of the droplet with a filter paper to remove the excess liquid and allowing the grid to dry. No staining of the samples was necessary. Images were analyzed using the ImageJ software package (version 1.52a) developed at the US National Institute of Health. X-ray photoelectron spectroscopy (XPS) spectra were collected with an AXIS ULTRA DLD XPS System with MONO AI source (Shimadzu Kratos. Japan). Microscopic imaging infrared spectroscopy (MIR) was conducted on NicoletiN10MX. Samples were scraped off from the substrate and scanned from 4000 to 400 cm^-1^ after focusing. X-ray diffraction (XRD) patterns were obtained on a D8 ADVANCE Da Vinci diffractometer equipped with Cu Kα radiation (40 kV, 40 mA). N_2_ adsorption-desorption isotherms were obtained at 77K on an Autosorb-IQ3 analyzer. Before measurements, the samples were degassed in a vacuum at 80 °C for 10 h. Thermogravimetric analysis (TGA) was performed on a Mettler Toledo TGA-SDTA851 analyzer (Switzerland) under air atmosphere from 25 to 600 °C with a heating rate of 10 °C min^-1^. The methanol oxidation process was monitored by on-line gas chromatography (on-line GC) with a thermal conductivity detector (TCD) (Shimazu GC-2014). Methanol oxidation products including CH_3_OH, CH_3_OCH_2_OCH_3_, HCOOCH_3_, HCHO, CH_3_OCH_3_, and H_2_O were analyzed with a Porapak T column. The gas sensing performances were evaluated on the MA1.0 measuring system (Narui Corp Ltd., China).

**Preparation of PFS-*b*-P2VP Micelle Seeds**

Typically, 5 mg of PFS_44_-*b*-P2VP_526_ powder was firstly added to 10 mL of isopropanol and stirred at 80 °C until completely dissolved. The resulting bright yellow transparent solution (0.5 mg/mL) was then cooled to room temperature and aged overnight to allow the formation of long polydisperse cylindrical micelles. Then probe ultrasonic processor (150 W) was used to fragmentize the resulting cylindrical micelles in an ice-water bath at 0 °C for 60 min to form short micelle seeds.

**Decoration of PFS-*b*-P2VP Micelle Seeds on Silicon Wafer**

The silicon wafer (1 × 1 cm) was firstly boiled in a mixture of 3.5 mL of 98% H_2_SO_4_ and 1.5 mL of 30% H_2_O_2_ (7/3, v/v) to allow the formation of hydroxyl functional groups on the surface, and was then sonicated alternatively in acetone and water for further cleaning. In a typical process, 20 µL of a solution of the PFS_44_-*b*-P2VP_526_ seeds (0.5 mg/mL in isopropanol) was dripped onto the surface of the treated silicon wafer and dried in the air. The resulting sample was then rinsed with isopropanol to remove the excess, poorly immobilized seeds.

**Decoration of PFS-*b*-P2VP Micelle Seeds on Ceramic Tube**

In a typical process, the miniature ceramic tube (length = 4 mm; external diameter = 1 mm) was firstly ultrasonically cleaned alternatively with water and ethanol. The treated ceramic tubes was then repeatedly soaked in a solution of the PFS_44_-*b*-P2VP_526_ seeds (0.5 mg/mL in isopropanol) by three times and allowed to dry in the air. The resulting sample was eventually rinsed with isopropanol to remove the excess, poorly immobilized seeds.

**Growth of PFS-*b*-P2VP Soft Nanobrushes**

Typically, a PFS_44_-*b*-P2VP_526_ micelle seed-coated silicon wafer was placed in 1 mL of isopropanol. Then, a solution of the PFS_24_-*b*-P2VP_314_ unimers (10 mg/mL in THF) was added and the mixture was vibrated for 2 hours using a shaker. The resulting sample was finally rinsed with isopropanol to remove the excess unimers. PFS-*b*-P2VP soft nanobrushes were also grown on the ceramic tubes following a similar procedure.

**Supplementary Figures**


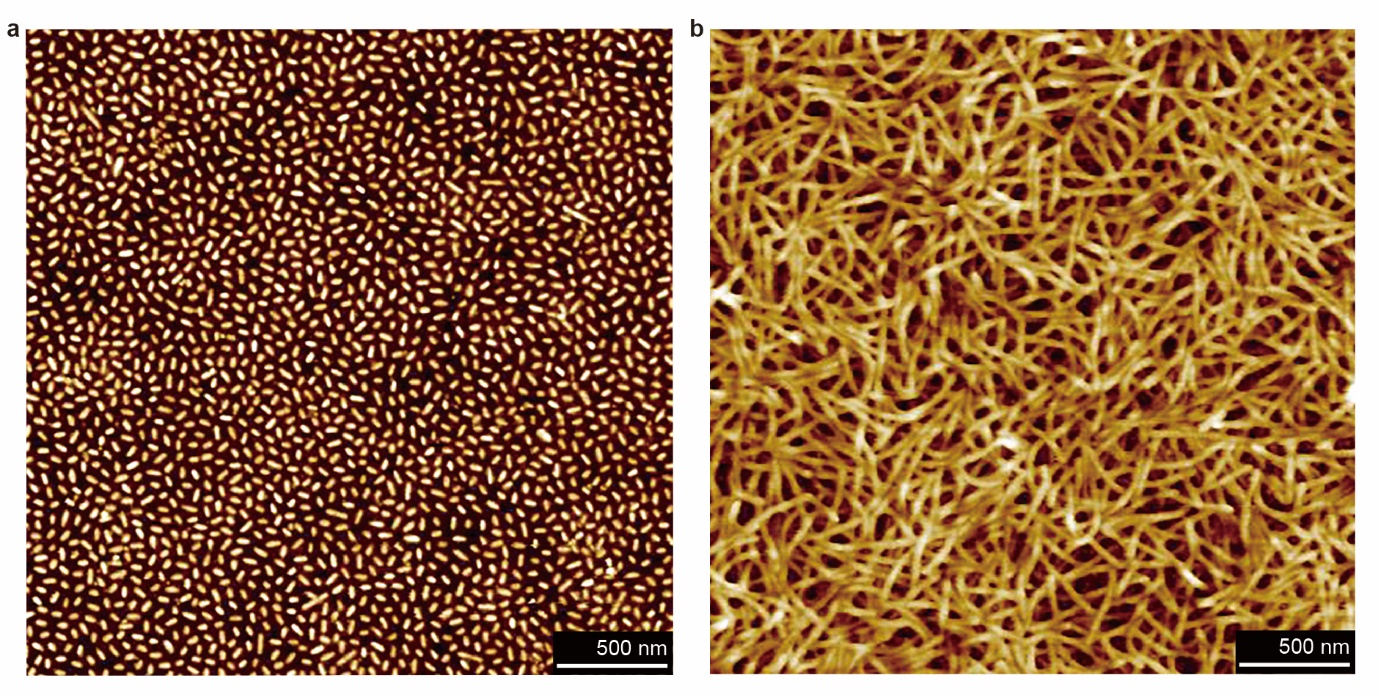


**Supplementary Figure 1. Growth of PFS-*b*-P2VP soft nanobrush on silicon wafer. a)** AFM height image of PFS_44_-*b*-P2VP_526_ micelle seeds (*L*_n_ = 65 nm, *L*_w_ = 68 nm, *L*_w_/*L*_n_ = 1.05) coated on a silicon wafer. **b)** AFM height image of soft nanobrush prepared by adding 6 μL of a THF solution of PFS_24_-*b*-P2VP_314_ unimers (10 mg/mL) to the PFS_44_-*b*-P2VP_526_ micelle seed-coated silicon wafer (soaked in 1 mL of isopropanol).


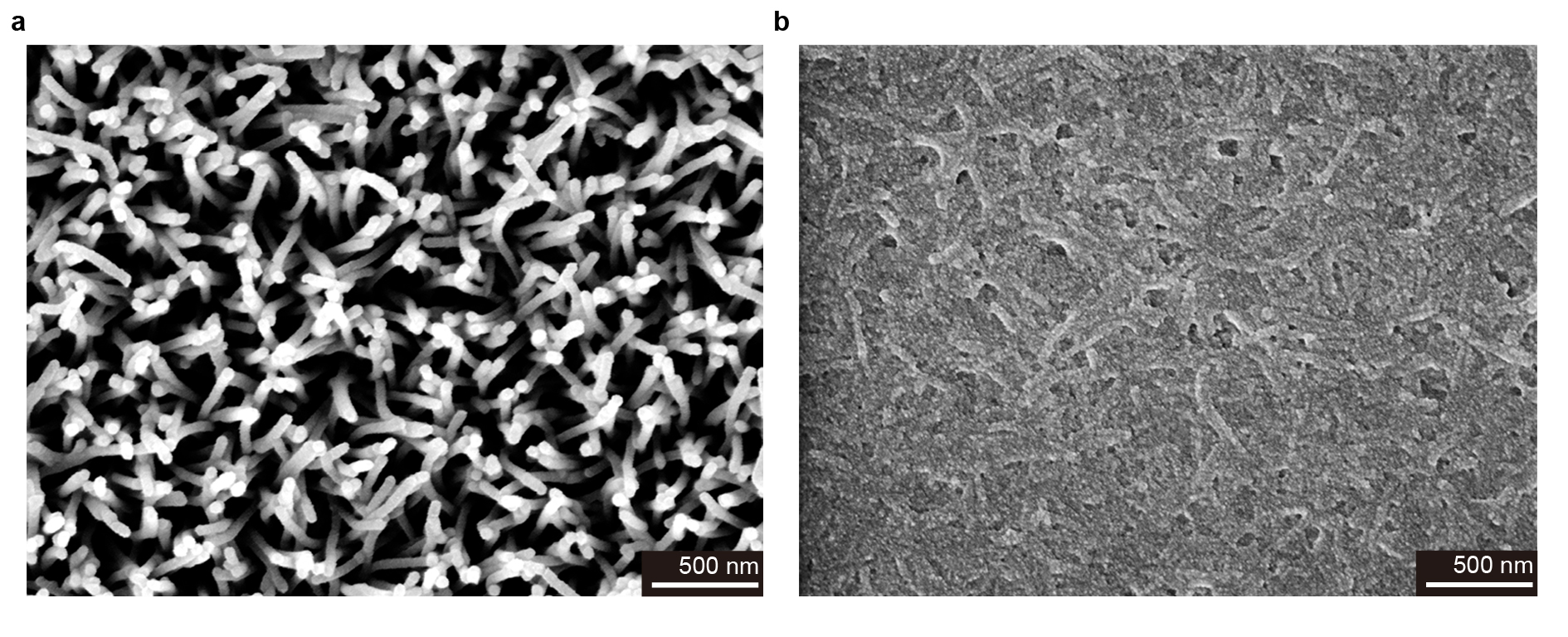


**Supplementary Figure 2. Morphology comparison of samples fabricated by alternating immersion and a one-pot strategy.** SEM images of **a)** MIL-100 (Fe) nanoarray formed by alternating immersion and **b)** irregular composite obtained by a one-pot method.


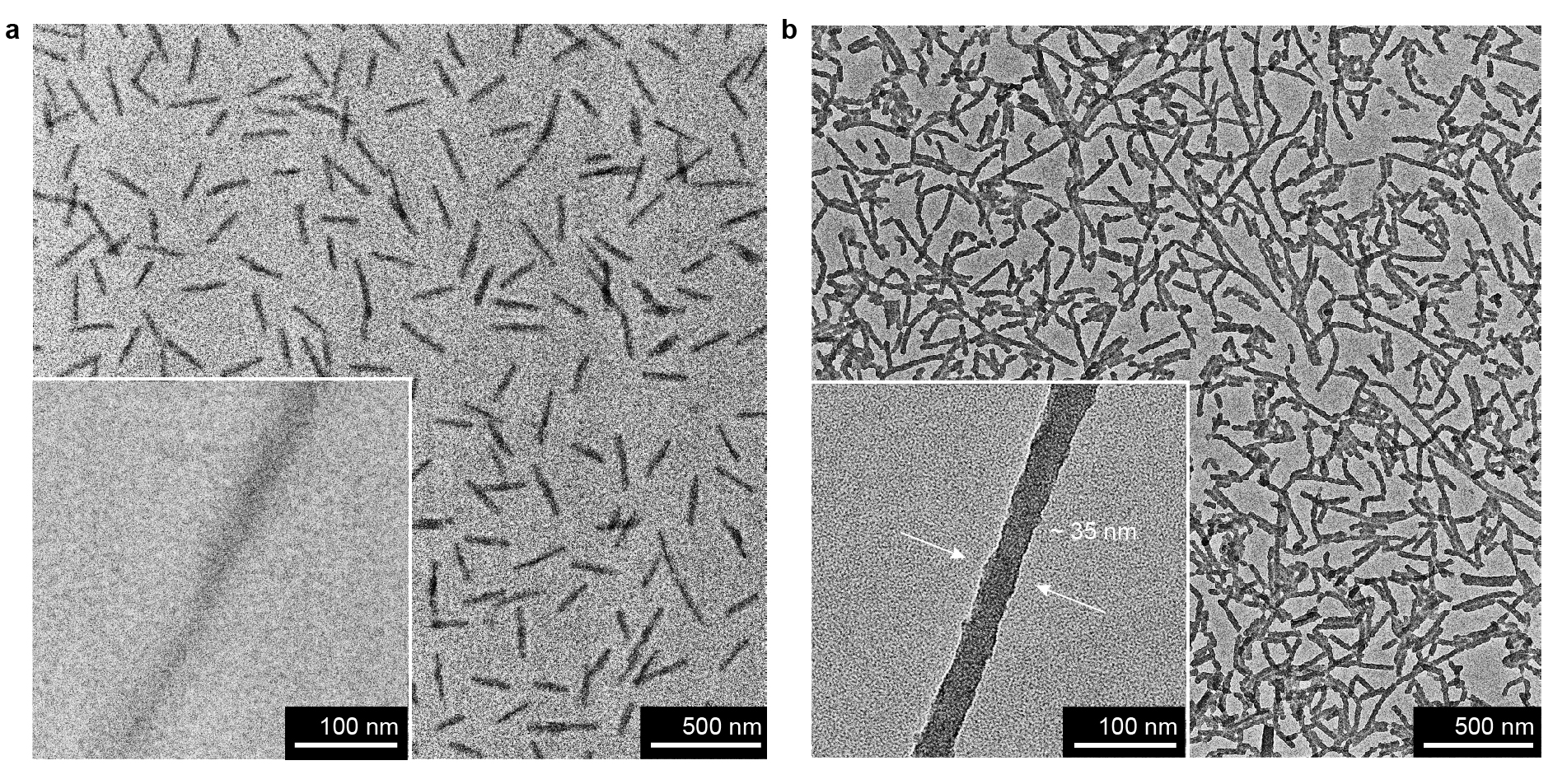


**Supplementary Figure 3. Morphology comparison between pristine soft nanobrush and MIL-100 (Fe) nanoarray.** TEM images of **a)** pristine soft nanobrush and **b)** MIL-100 (Fe) nanoarray corresponding to the sample shown in Fig. 2b. The samples were removed from the silicon wafer via ultrasonication.


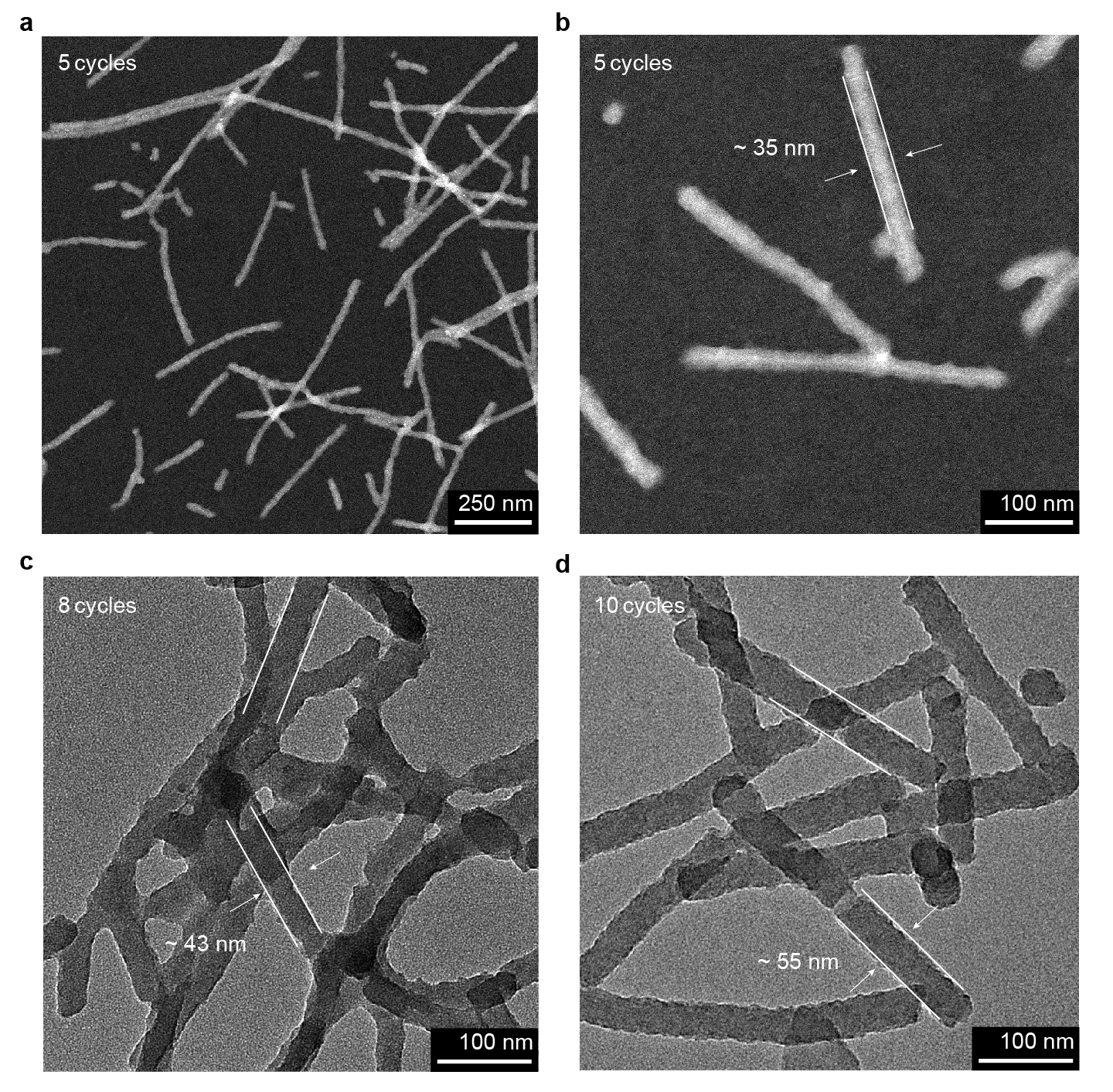


**Supplementary Figure 4.** **Thickness modulation of MIL-100 (Fe) nanoarrays. a, b)** HAADF-STEM images of the MIL-100 (Fe) nanoarray corresponding to the sample shown in Fig. 2b. **c, d)** TEM images of the MIL-100 (Fe) nanoarrays obtained by repeatably immersing the soft nanobrush-coated silicon wafer in ethanol solutions of Fe^3+^ and H_3_BTC for (c) eight and (d) ten cycles. The samples were removed from the silicon wafer via ultrasonication.


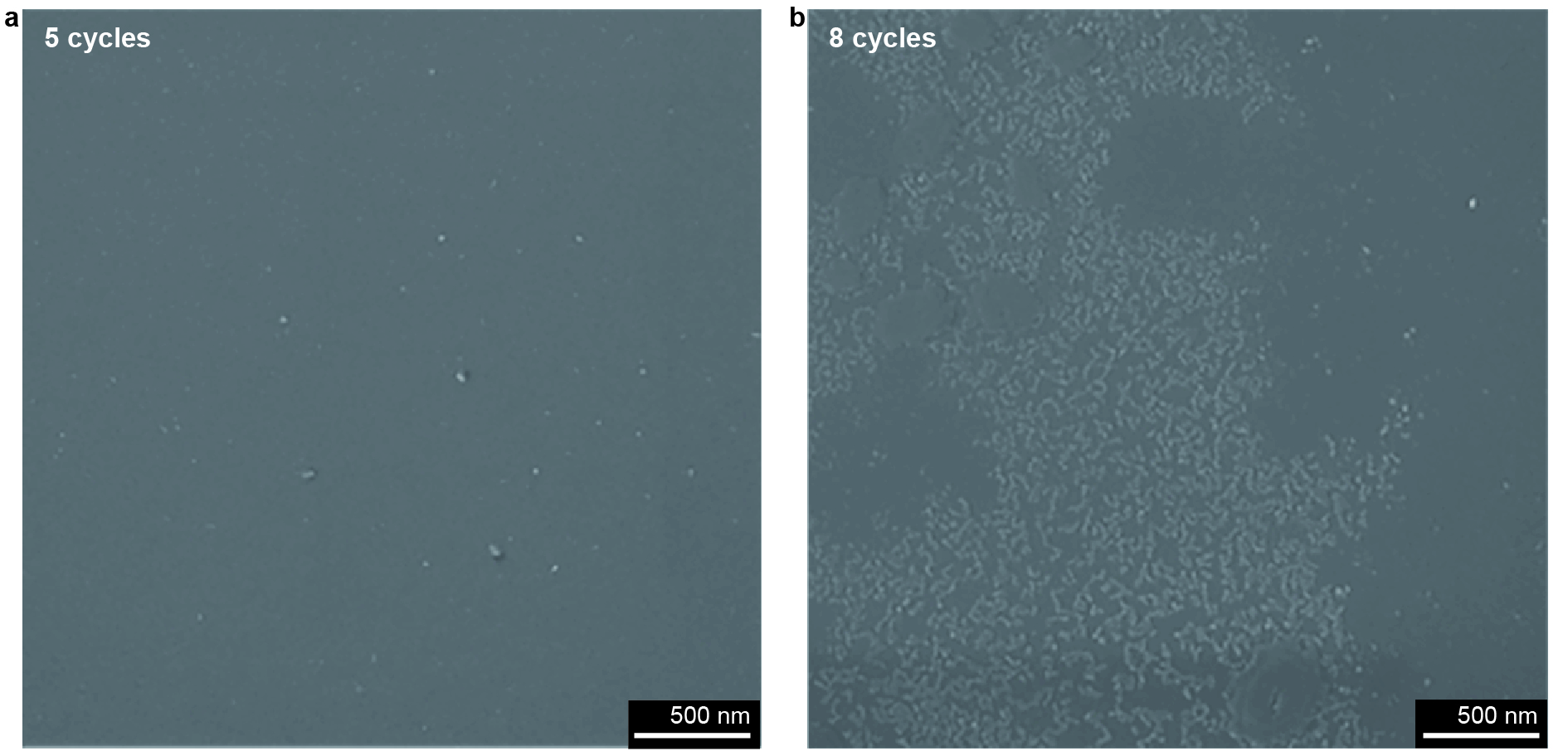


**Supplementary Figure 5. Control experiments for the growth of MIL-100 (Fe) on naked silicon wafers.** SEM images of silicon wafers after being repeatedly immersed in ethanol solutions of Fe^3+^ and H_3_BTC for **a)** five and **b)** eight cycles.


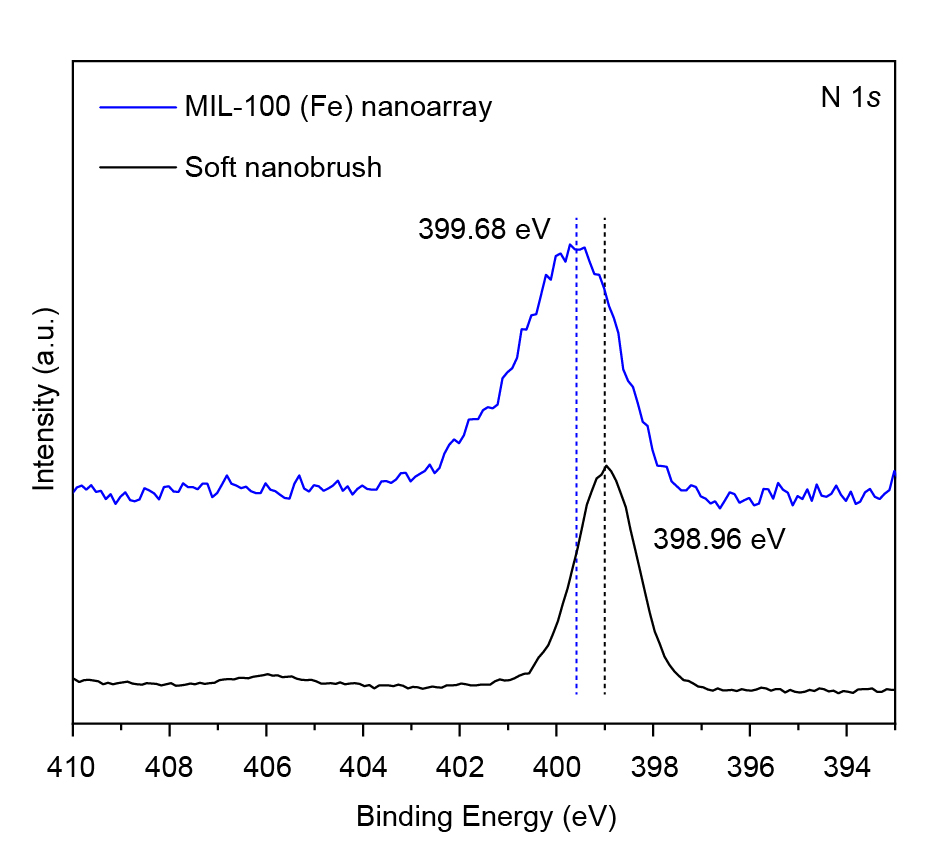


**Supplementary Figure 6.** XPS spectra of the MIL-100 (Fe) nanoarray shown in Fig. 2b and the corresponding soft nanobrush template.


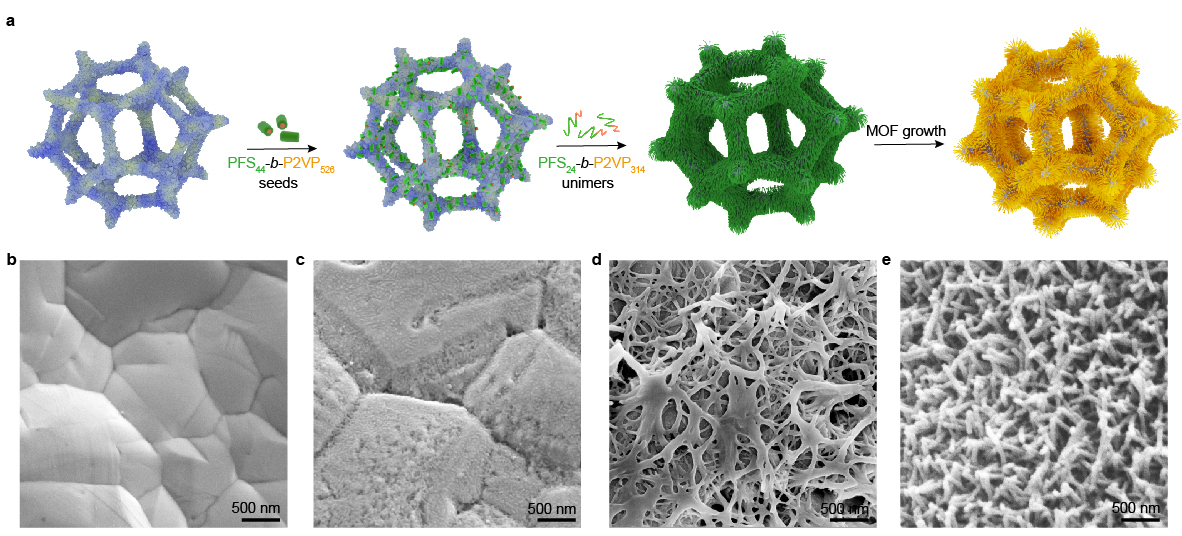


**Supplementary Figure 7. Growth of MIL-100 (Fe) nanoarray on Ni foam. a)** Schematic illustration of the fabrication process. **b-e)** SEM images of pristine Ni foam (b), micelle seed-coated Ni foam (c), soft nanobrush grown on Ni foam (d), and MIL-100 (Fe) nanoarray (16 μL) grown on Ni foam (e). Size of Ni foam: 1 × 1 × 0.05 cm**.**


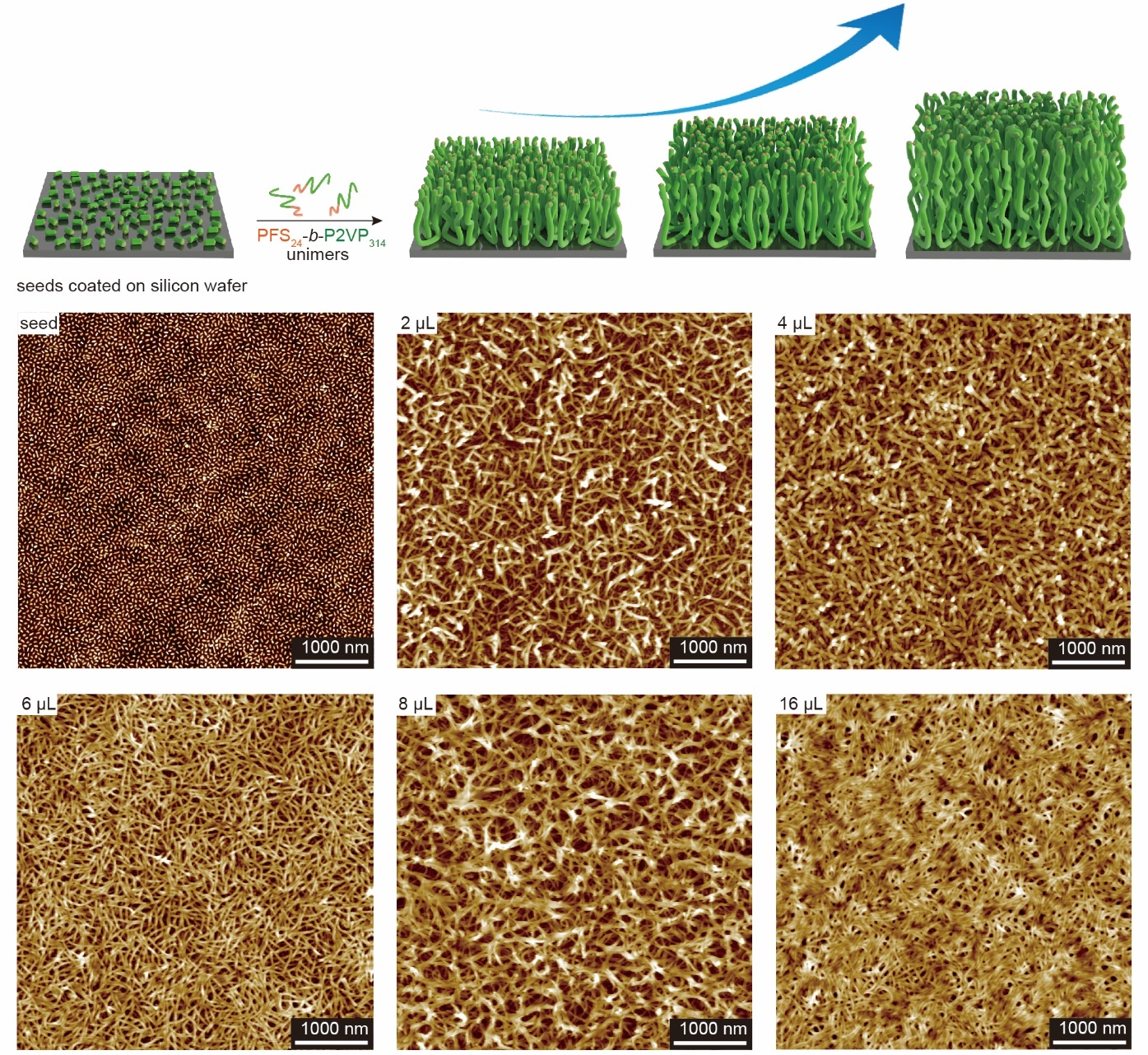


**Supplementary Figure 8.** **Length modulation of PFS_24_-*b*-P2VP_314_ soft nanobrushes. Top:** Scheme illustration of the growth process. **Below:** AFM height images of PFS_24_-*b*-P2VP_314_ soft nanobrushes formed with the addition of 2, 4, 6, 8 and 16 μL of a THF solution of PFS_24_-*b*-P2VP_314_ unimers (10 mg/mL in THF) to the PFS_44_-*b*-P2VP_526_ micelle seed-coated silicon wafers (soaked in 1 mL of isopropanol).


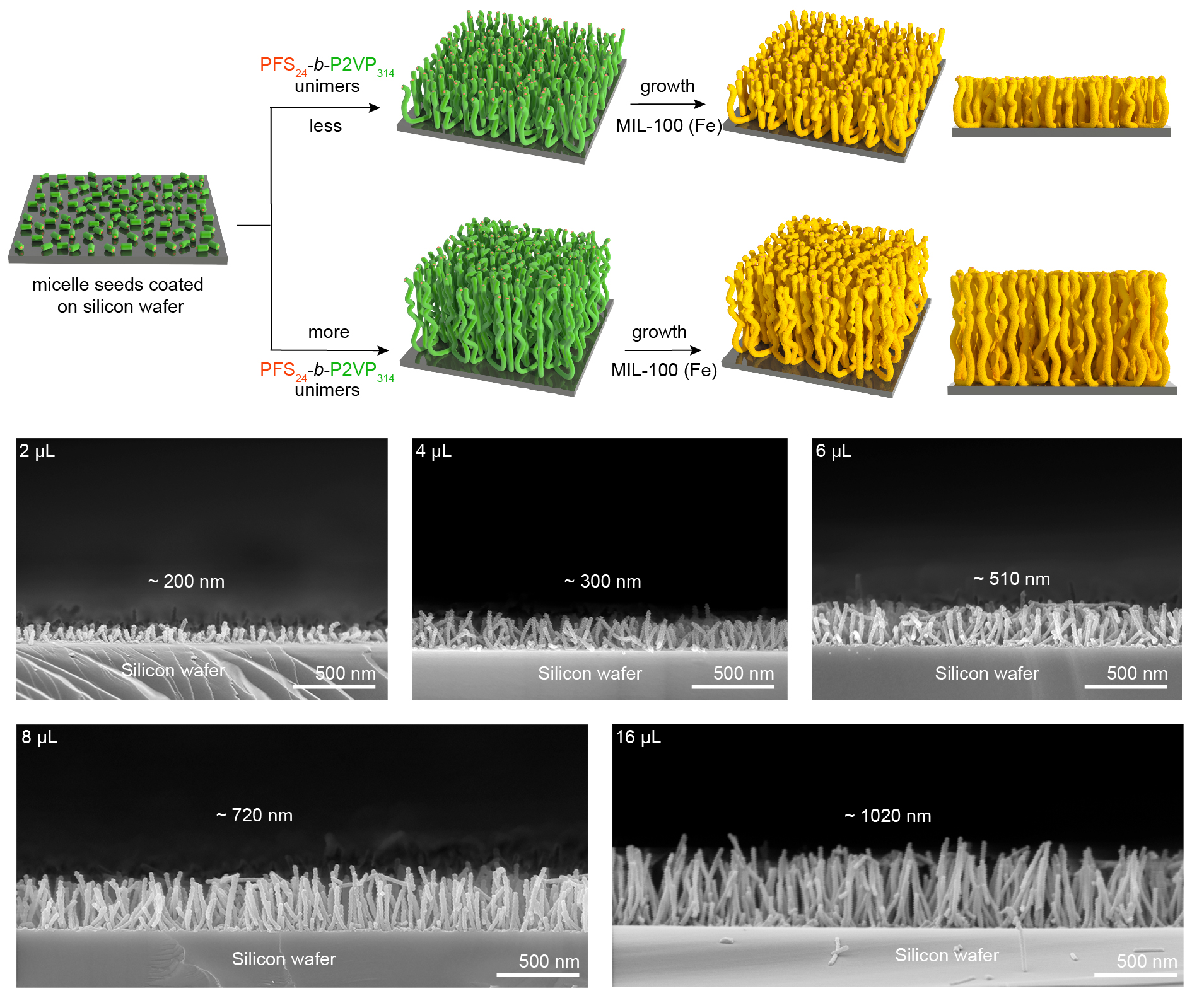


**Supplementary Figure 9. Height regulation of MIL-100 (Fe) nanoarrays. Top:** Scheme illustration of the fabrication process. **Below:** Cross-sectional SEM images of the MIL-100 (Fe) nanoarrays directed by the soft nanobrushes formed with the addition of 2, 4, 6, 8 and 16 μL of a THF solution of PFS_24_-*b*-P2VP_314_ unimers (10 mg/mL in THF).


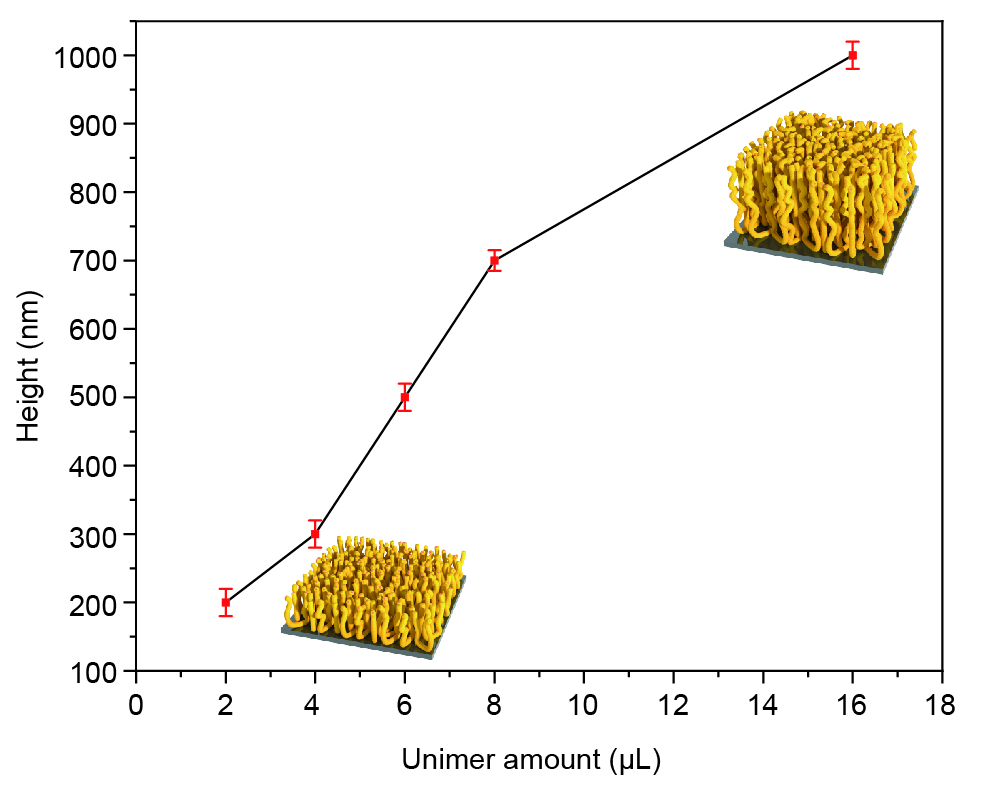


**Supplementary Figure 10**. **Plot of the height of the MIL-100 (Fe) nanoarray against the volume of the unimer solution added for the growth of soft nanobrushes.** The heights of the MIL-100 (Fe) nanoarrays corresponding to the samples shown in Fig. 3b were measured from ≥5 locations (≥5 cross-sectional SEM images were captured) for each sample.


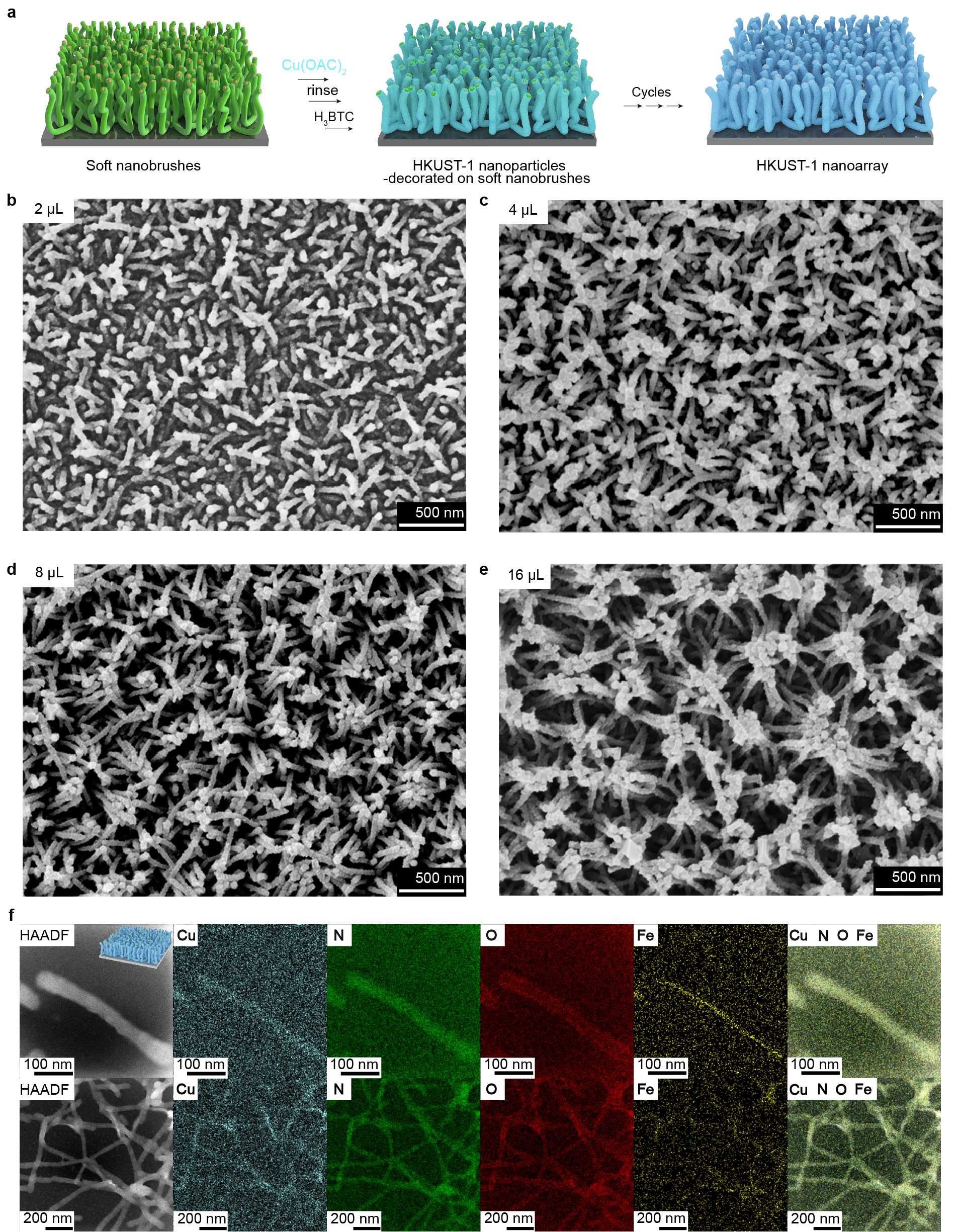


**Supplementary Figure 11. Fabrication of HKUST-1 nanoarrays. a)** Schematic illustration of the fabrication process. **b-e)** SEM images of the HKUST-1 nanoarrays directed by the soft nanobrushes formed by adding 2 (b), 4 (c), 8 (d), and 16 μL (e) of a solution of PFS_24_-*b*-P2VP_314_ unimers (10 mg/mL in THF). **f)** HAADF-STEM images and elemental mapping of the nanorods of the HKUST-1 nanoarray corresponding to the sample shown in Figure 4b, which were removed from the silicon wafer via ultrasonic treatment.


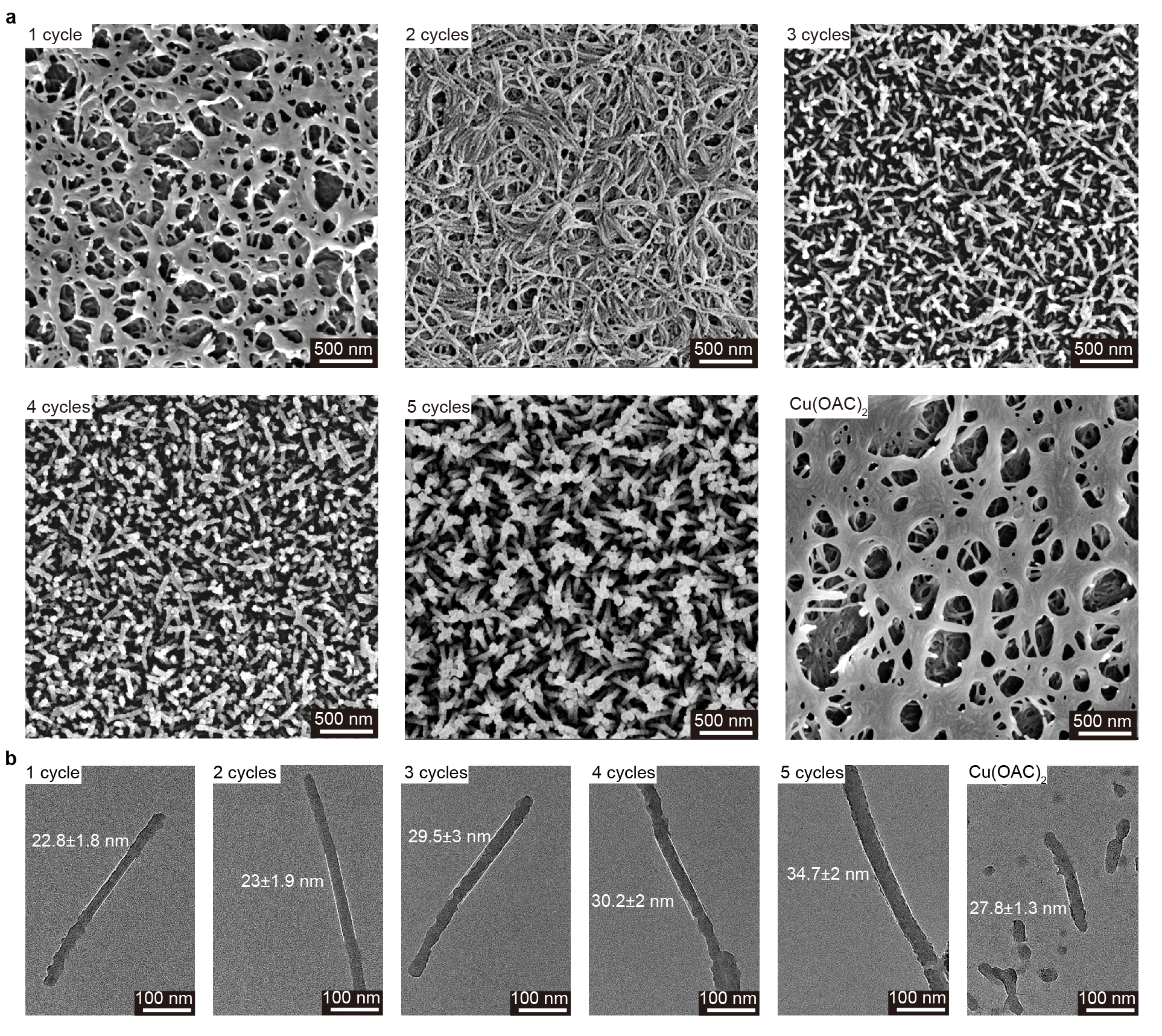


**Supplementary Figure 12.** SEM **a)** and TEM images **b)** of soft nanobrushes after alternately immersing in ethanol solutions of Cu(CH_3_COO)_2_ and H_3_BTC for 1 to 5 cycles, and immersing in an ethanol solution of Cu(CH_3_COO)_2_ alone.


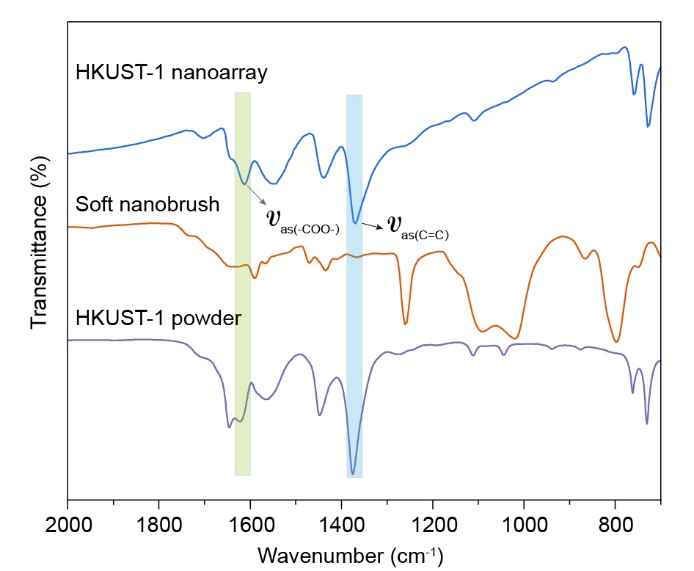


**Supplementary Figure 13.** FT-IR spectra of HKUST-1 nanoarray (16 µL), soft nanobrush and HKUST-1 powder. The peaks at 1587, 1565, 1469 cm^-1^ originate from the stretching vibrations of the double bonds in the pyridyl groups of PFS_24_-*b*-P_2_VP_314_, while the peaks at 1623 and 1373 cm^-1^ can be attributed to the -COO- and C=C vibrations of HKUST-1.


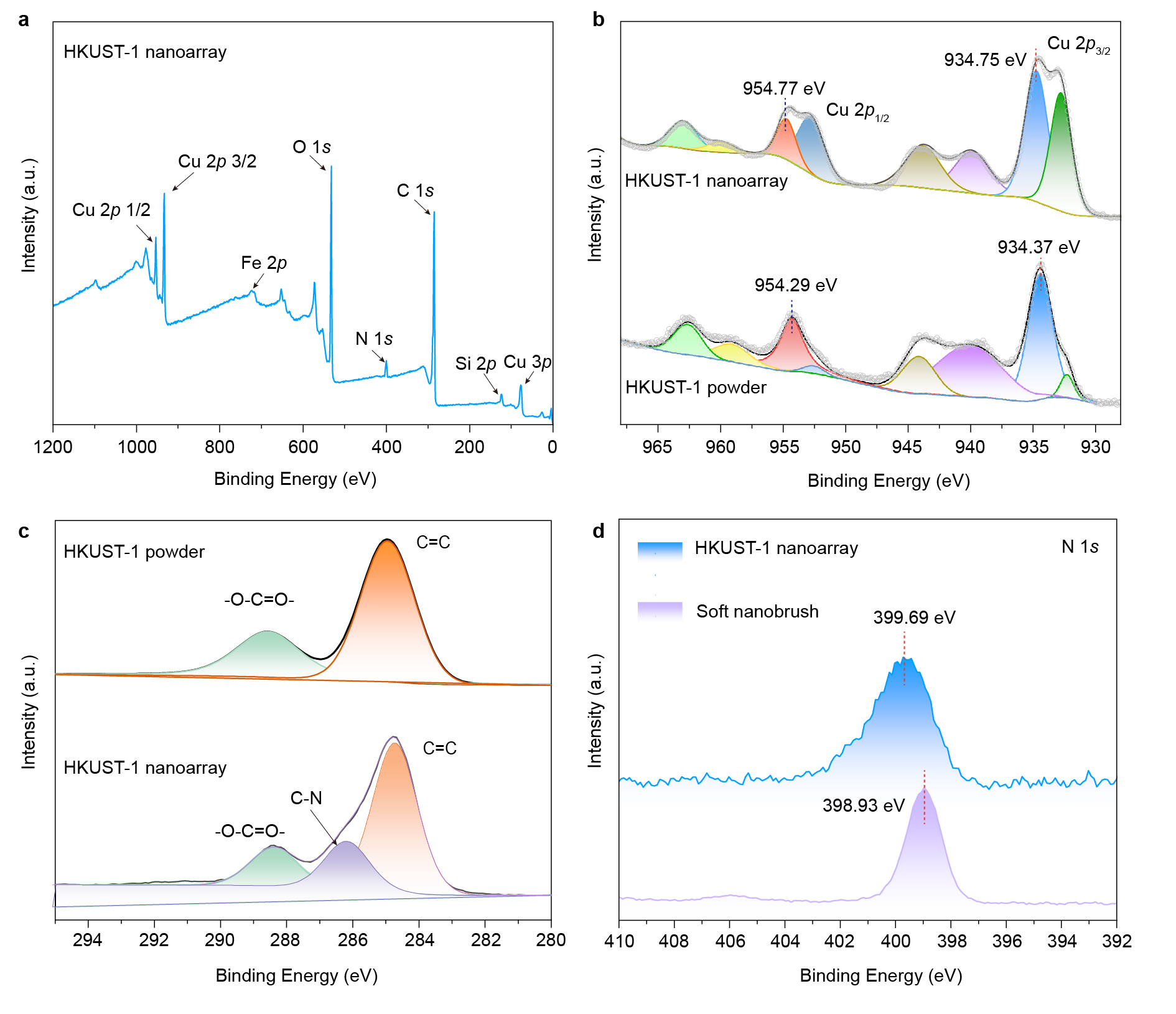


**Supplementary Figure 14.** **a)** Survey scan XPS spectra of HKUST-1 nanoarray (16 µL). **b)** Cu 2*p* and **c)** C 1*s* regions of HKUST-1 nanoarray (16 µL) and HKUST-1 powder. **d)** N 1*s* regions of HKUST-1 nanoarray (16 µL) and soft nanobrush.


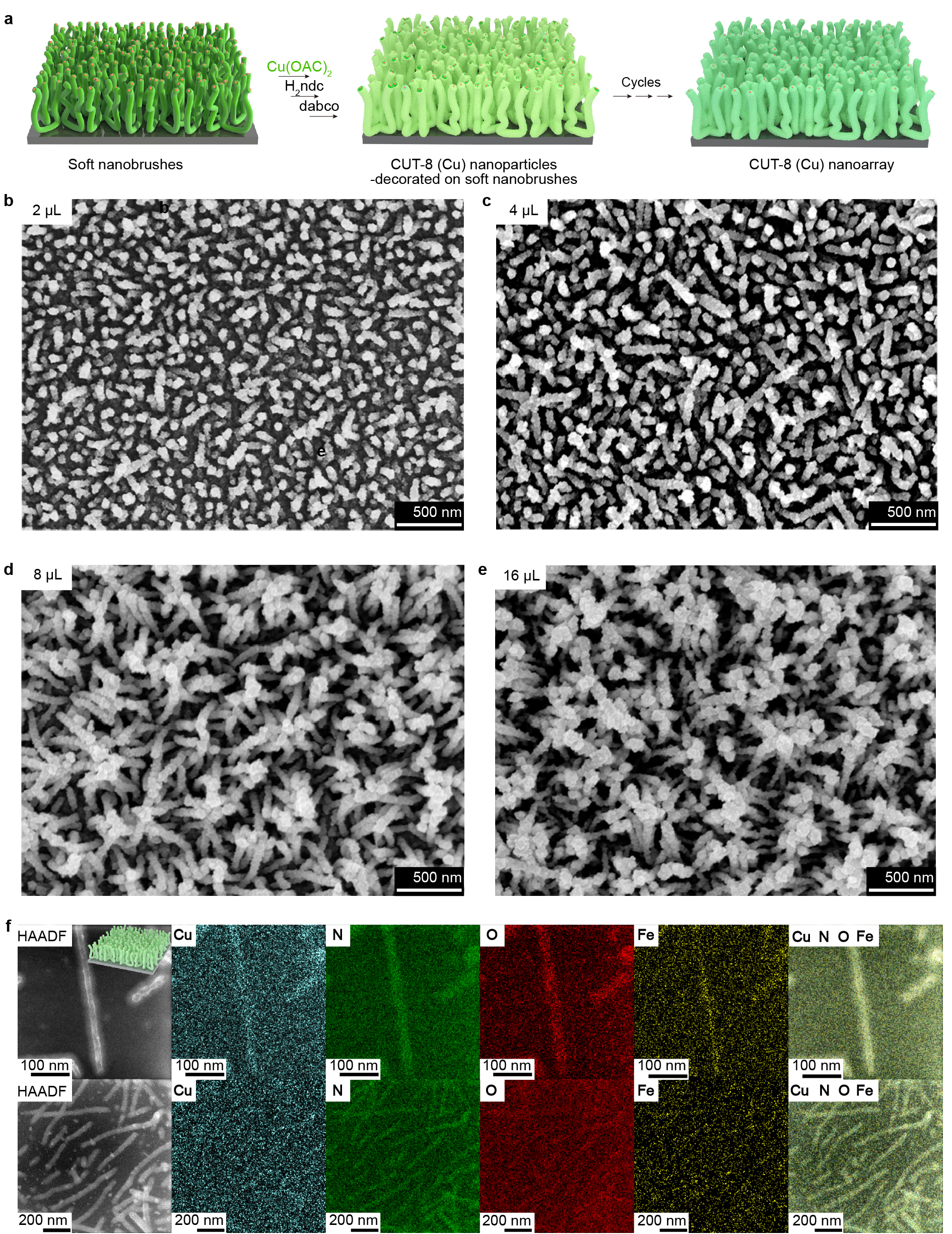


**Supplementary Figure 15. Fabrication of CUT-8 (Cu) nanoarrays. a)** Schematic illustration of the fabrication process. **b-e)** SEM images of the CUT-8 (Cu) nanoarray directed by the soft nanobrushes formed by adding 2 (b), 4 (c), 8 (d), and 16 μL (e) of a solution of PFS_24_-*b*-P2VP_314_ unimers (10 mg/mL in THF). **f)** HAADF-STEM images and elemental mapping of the nanorods of the CUT-8 (Cu) nanoarray corresponding to the sample shown in Figure 4d, which were removed from the silicon wafer via ultrasonic treatment.

**Supplementary Table 1** Catalytic performance of MIL-100 (Fe) nanoarrays for oxidation of methanol*^a^*


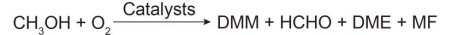


| Catalysts | Temp. (°C) | Conv. (%) | Reaction rate (mol·g^-1^·h^-1^) | Selectivity (%) | | | | TOF*^b^* (h^-1^) |
| --- | --- | --- | --- | --- | --- | --- | --- | --- |
|  |  |  |  | DMM HCHO DME MF | | | |  |
| MIL-100 (Fe) nanoarray (2 µL) | 160 | 32.39 | 2.483 | 0 | 78.0 | 2.4 | 19.6 | 125.76 |
| MIL-100 (Fe) nanoarray (6 µL) | 160 | 35.27 | 2.283 | 0 | 77.0 | 0 | 23 | 257.15 |
| MIL-100 (Fe) nanoarray (16 µL) | 160 | 55.95 | 4.333 | 0 | 72.7 | 0 | 27 | 216.25 |
| MIL-100 (Fe) powder | 160 | 21.23 | 0.000066 | 54.6 | 21.4 | 8.3 | 15.7 | 0.0355 |
| Supported MIL-100 (Fe) powder | 160 | 18.52 | 0.0008 | 67.5 | 20.6 | 1.1 | 10.8 | 0.773 |

*^a^*Reaction condition: methanol was introduced into the reactor with a liquid hourly space velocity (LHSV) of 0.19 h^-1^ by a constant flow pump; O_2_ was used as the oxidant and the gas hourly space velocity (GHSV) was 2832 mL/h/g. The reaction rate was calculated as the dynamic behavior over a 1 h period. Conversion and selectivity were determined by GC/MS. *^b^*Initial TOF values [TOF = mol FA/mol catalyst × time (h)] were calculated at ~20% of conversion achieved.


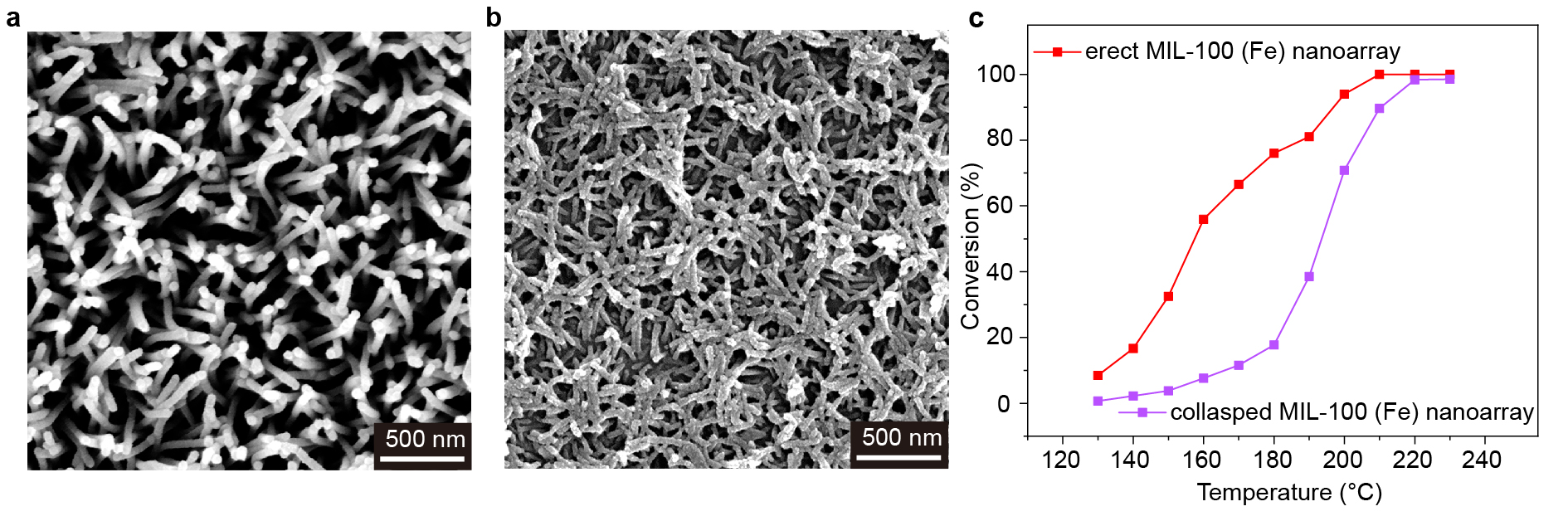


**Supplementary Figure 16. Effect of morphology on catalytic performance for MIL-100 (Fe) nanoarrays.** SEM images of **a)** erect and **b)** collapsed MIL-100 (Fe) nanoarrays, where the collapsed nanoarray was obtained by direct drying in air. **c)** Catalytic performance of erect and collapsed MIL-100 (Fe) nanoarrays.


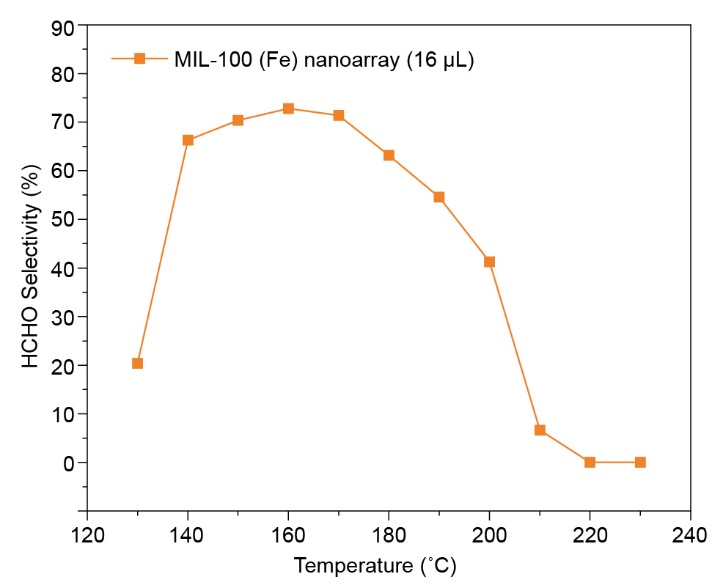


**Supplementary Figure 17. Temperature dependence of HCHO selectivity for the MIL-100 (Fe) nanoarray (16 µL).** The selectivity of HCHO increased at first and then gradually decreased with temperature, where it reached a maximum value of 72.7 % at 160 °C. It should be noted that HCHO would be further converted into CO_2_ under higher temperatures.


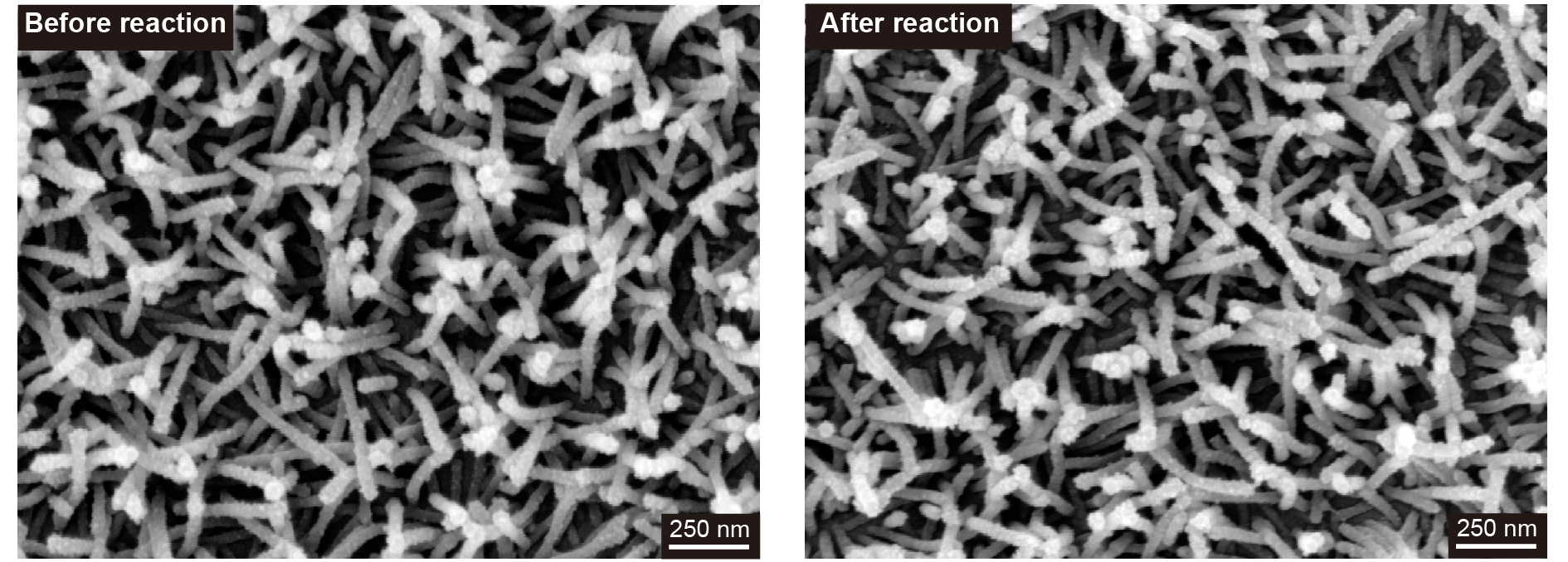


**Supplementary Figure 18.**  **Morphology of MIL-100 (Fe) nanoarray before and after reaction.** The MIL-100 (Fe) nanoarray (16 µL) remained erect after the catalytic reaction.


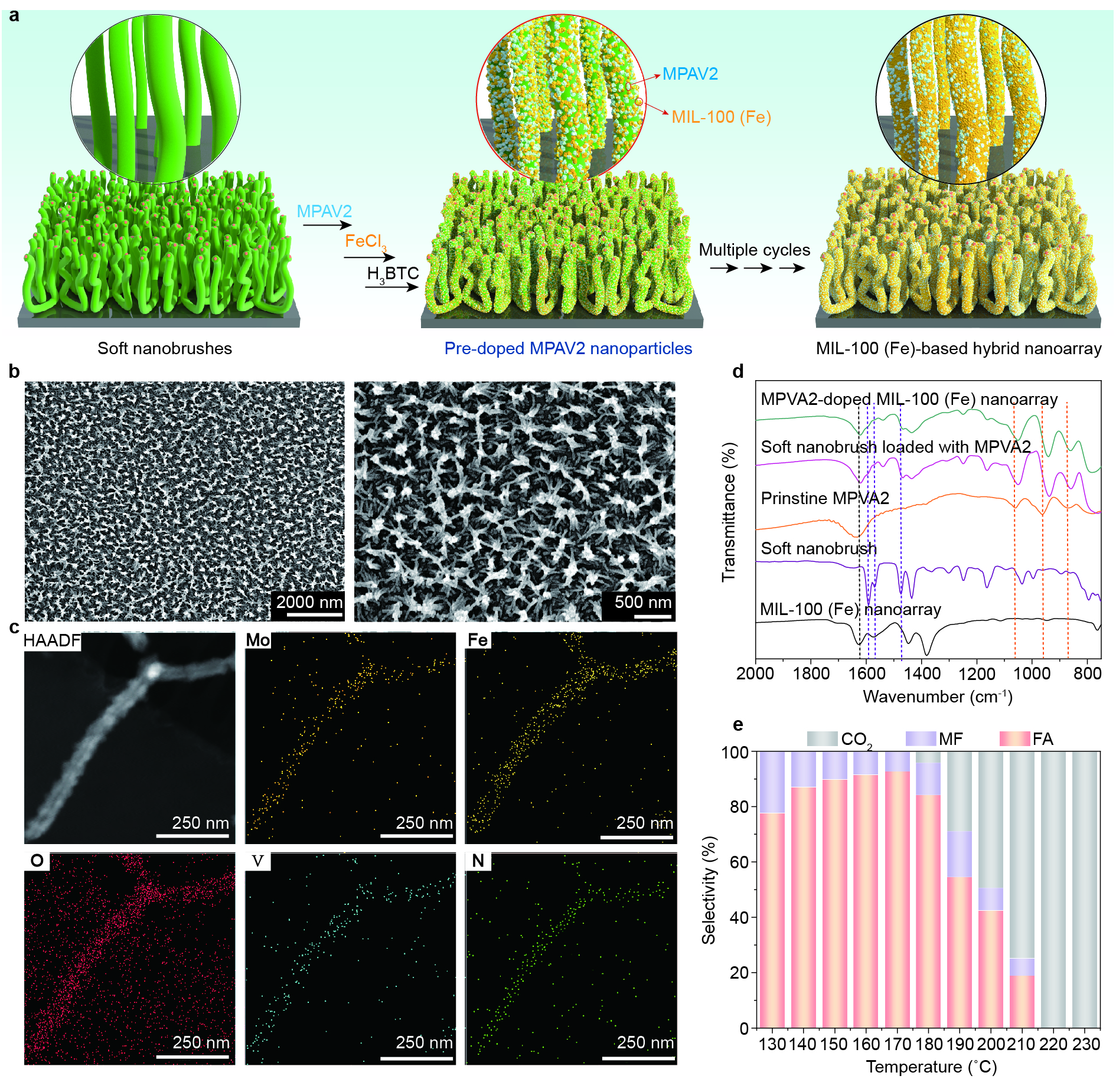


**Supplementary Figure 19. Fabrication and catalytic performance of MPAV2-doped MIL-100 (Fe) nanoarray (16 µL). a)** Schematic illustration of the fabrication process. **b)** SEM images of the MPAV2 (21.5 wt%)-doped MIL-100 (Fe) nanoarray (16 µL). **c)** HAADF-STEM images and elemental mapping of a nanorod of the MPAV2 (21.5 wt%)-doped MIL-100 (Fe) nanoarray shown in (b), which was removed from the silicon wafer via ultrasonication. **d)** FT-IR spectrum of MIL-100 (Fe) nanoarray (16 µL), soft nanobrushes, pristine MPAV2, soft nanobrush loaded with MPAV2, and MPAV2-doped MIL-100 (Fe) nanoarray (16 µL), respectively. The peaks of MPAV2-doped MIL-100 (Fe) nanoarray (16 µL) in the range of 800-1100 cm^-1^ can be attributed to the stretching vibration of Mo-O and V-O of MPAV2 and the peaks at 1587, 1565, 1469 cm^-1^ would originate from the stretching vibrations of the double bonds in the pyridyl groups of the PFS_24_-*b*-P_2_VP_314_, while the peaks at 1627 and 1444 cm^-1^ can be attributed to C-H and Fe-O vibrations of MIL-100 (Fe). **e)** Product selectivity for the oxidation of methanol catalyzed by the MPAV2 (21.5 wt%)-doped MIL-100 (Fe) nanoarray (16 µL) at different temperatures.

**Supplementary Table 2** Catalytic performance of MIL-100 (Fe) nanoarrays and previously reported Fe-based catalysts

| Catalysts | Temp. (°C) | | 1. (Mpa ) | Conv. (%) | | Sel._HCHO_  (%) | Yield (%) | Reference |
| --- | --- | --- | --- | --- | --- | --- | --- | --- |
| MoO_x_/Fe_2_O_3_ | 175 | 0.1 | | | 50 | 89.0 | 44.5 | 4 |
| VO_x_/Fe_2_O_3_ | 230 | 0.1 | | | 50 | 53.0 | 26.5 | 5 |
| α-Fe_2_O_3_ | 300 | 0.1 | | | 65 | 5.6 | 3.6 | 6 |
| Al-doped Fe_2_O_3_ | 275 | 0.1 | | | 50 | 4.6 | 2.3 | 7 |
| FeAlVO_4_ | 350 | 0.1 | | | 95 | 88.0 | 83.6 | 8 |
| Fe_2.50(1−_*_z_*_/3)_V_0.20(1−_*_z_*_/3)_Mo_0.30(1−z/3)□_*_z_*O_4_ | 300 | 0.1 | | | 84 | 86.0 | 72.2 | 9 |
| FeVO_4_ | 230 | 0.1 | | | 81 | 83.0 | 67.2 | 10 |
| HDS-MoFeO | 240 | 0.1 | | | 30.7 | 78.8 | 24.2 | 11 |
| Fe_2_(MoO_4_)_3_/MoO_3_ | 300 | 0.1 | | | 100 | 80.0 | 80 | 12 |
| MIL-100 (Fe) nanoarray (16 µL) doped with MPAV2 (21.5%) | 160 | 0.1 | | | 50.2 | 92.5 | 46.2 | **This work** |


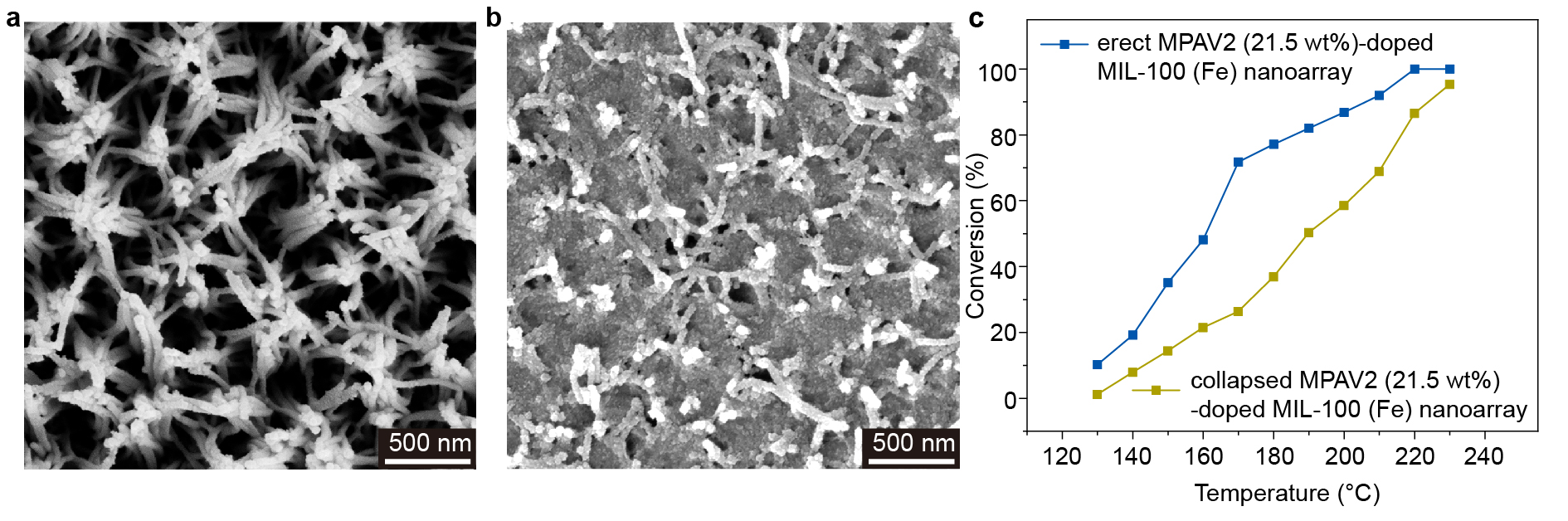


**Supplementary Figure 20. Effect of morphology on catalytic performance for MPAV2 (21.5 wt%)-doped MIL-100 (Fe) nanoarrays.** SEM images of **a)** erect and **b)** collapsed MPAV2 (21.5 wt%)-doped MIL-100 (Fe) nanoarrays, where the collapsed nanoarray was obtained by direct drying in air. **c)** Catalytic performance of erect and collapsed MPAV2 (21.5 wt%)-doped MIL-100 (Fe) nanoarray, respectively.

**Mechanism of methanol catalytic oxidation**

(i) The catalytic oxidation of CH_3_OH has been considered as an important industrial organic reaction and the reaction pathways have been proposed by Enrique Iglesia et al,^13^ which mainly involves primary transformation of CH_3_OH to form DME and HCHO, and secondary reactions of HCHO to form DMM and MF via hemiacetal (CH_3_OCH_2_OH) intermediates.^14,15^


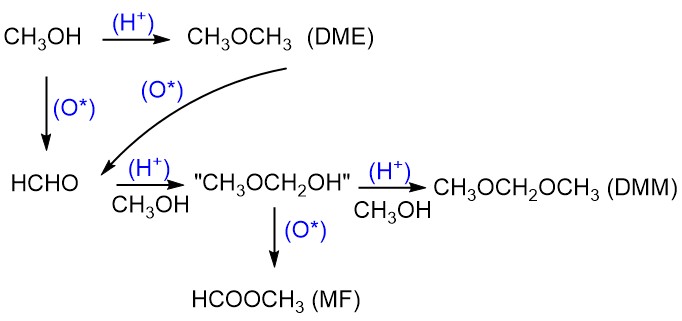


(ii) In our conditions, the main oxidation product was HCHO. It was postulated that in a typical pathway, CH_3_OH molecules are firstly adsorbed on the coordinately unsaturated Fe-sites of the MIL-100 (Fe) nanoarrays. Subsequently, oxidation dehydrogenation occurs and the hydrogens transfer from the O-H and C-H moieties of CH_3_OH to the iron oxide clusters.^16^ The resulting HCHO then desorbs from the iron oxide clusters and meanwhile the hydrogen atoms on the iron oxide clusters combined with gaseous oxygen to form H_2_O. On the other hand, the introduction of MPAV2^17,18^ further increased the HCHO selectivity and this was mainly attributed to the significant redox character of the VO_x_ units in the MPAV2 structure which can accelerate the oxidation dehydrogenation process of CH_3_OH.


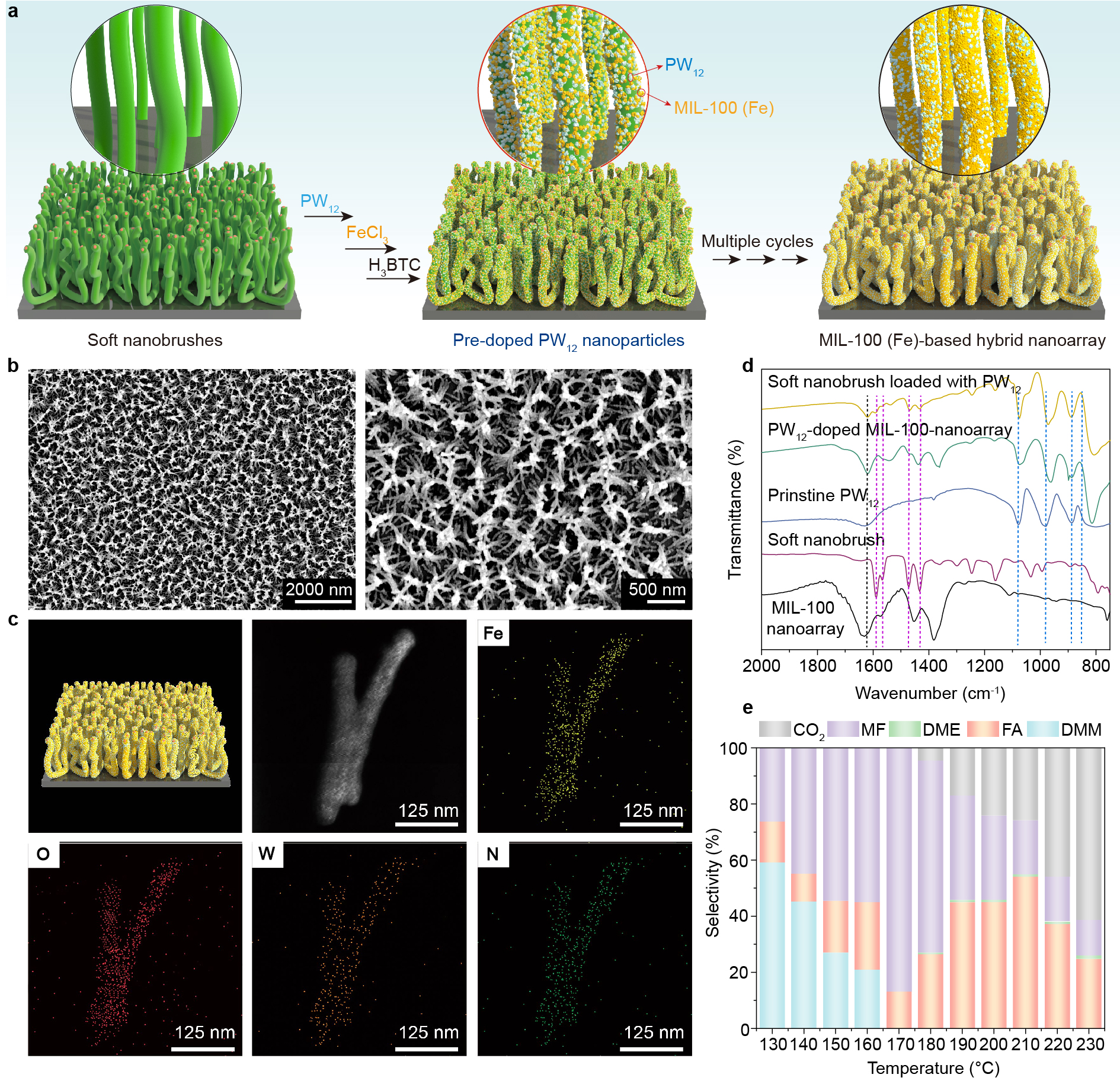


**Supplementary Figure 21. Fabrication and catalytic performance of PW_12_-doped MIL-100 (Fe) nanoarray (16 µL). a)** Schematic illustration of the fabrication process. **b)** SEM images of the PW_12_-doped MIL-100 (Fe) nanoarrays (16 µL). **c)** HAADF-STEM images and elemental mapping of a nanorod of the PW_12_-doped MIL-100 (Fe) nanoarrays (16 µL) shown in (b), which was removed from the silicon wafer via ultrasonication. **d)** FT-IR spectrum of the MIL-100 (Fe) nanoarrays (16 µL), the soft nanobrushes, the pristine PW_12_, the soft nanobrushes loaded with PW_12_, and the PW_12_-doped MIL-100 (Fe) nanoarrays (16 µL) removed from the silicon wafer, respectively. The peaks of MPAV2-doped MIL-100 (Fe) nanoarray (16 µL) in the range of 800-1100 cm^-1^ can be attributed to the stretching vibration of W-O of PW_12_ and the peaks at 1587, 1565, 1469 cm^-1^ would originate from the stretching vibrations of the double bonds in the pyridyl groups of the PFS_24_-*b*-P_2_VP_314_, while the peaks at 1627 and 1444 cm^-1^ can be attributed to C-H and Fe-O vibrations of MIL-100 (Fe). **e)** Product selectivity for the oxidation of methanol catalyzed by the PW_12_ (18.6 wt%)-doped MIL-100 (Fe) nanoarray (16 µL) at different temperatures.


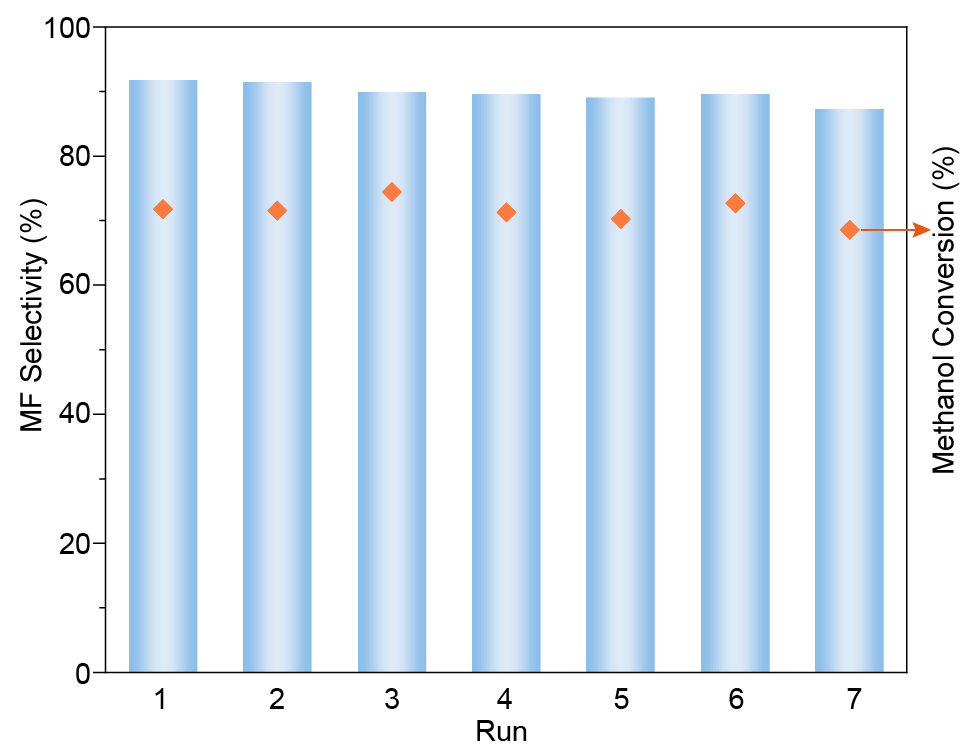


**Supplementary Figure 22.** **Recyclability of MPAV2 (21.5 wt%)-doped MIL-100 (Fe) nanoarray.** The catalytic activity of the MPAV2 (21.5 wt%)-doped MIL-100 (Fe) nanoarray was well retained after 7 cycles of methanol oxidation without any treatment, indicative of a high recyclability.


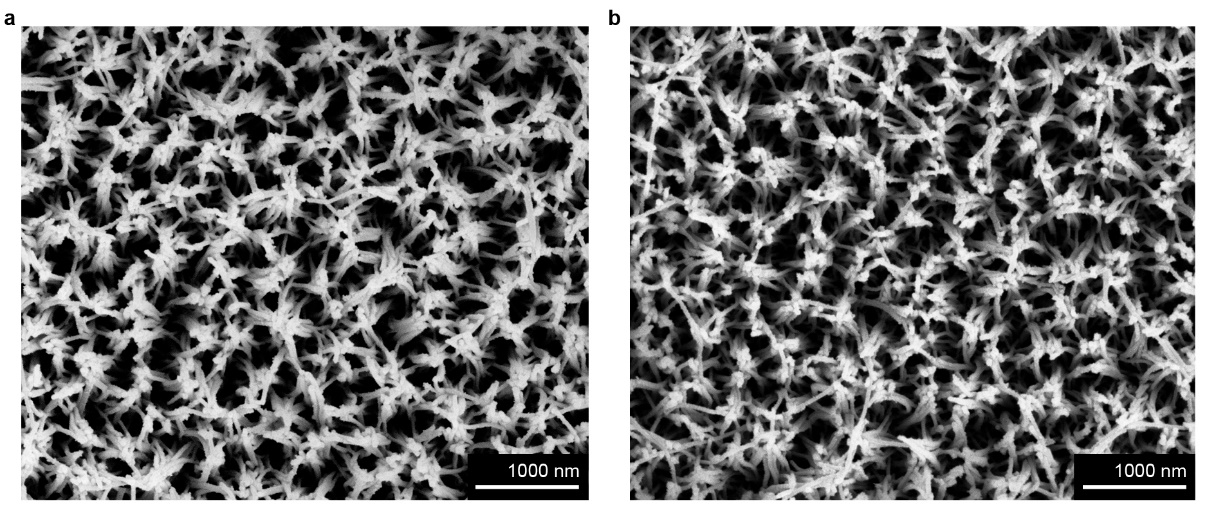


**Supplementary Figure 23.** **Morphology resistance of MPAV2 (21.5 wt%)-doped MIL-100 (Fe) nanoarray over the catalytic reaction.** SEM images of the MPAV2 (21.5 wt%)-doped MIL-100 (Fe) nanoarray before **a)** and after **b)** the catalytic reaction.


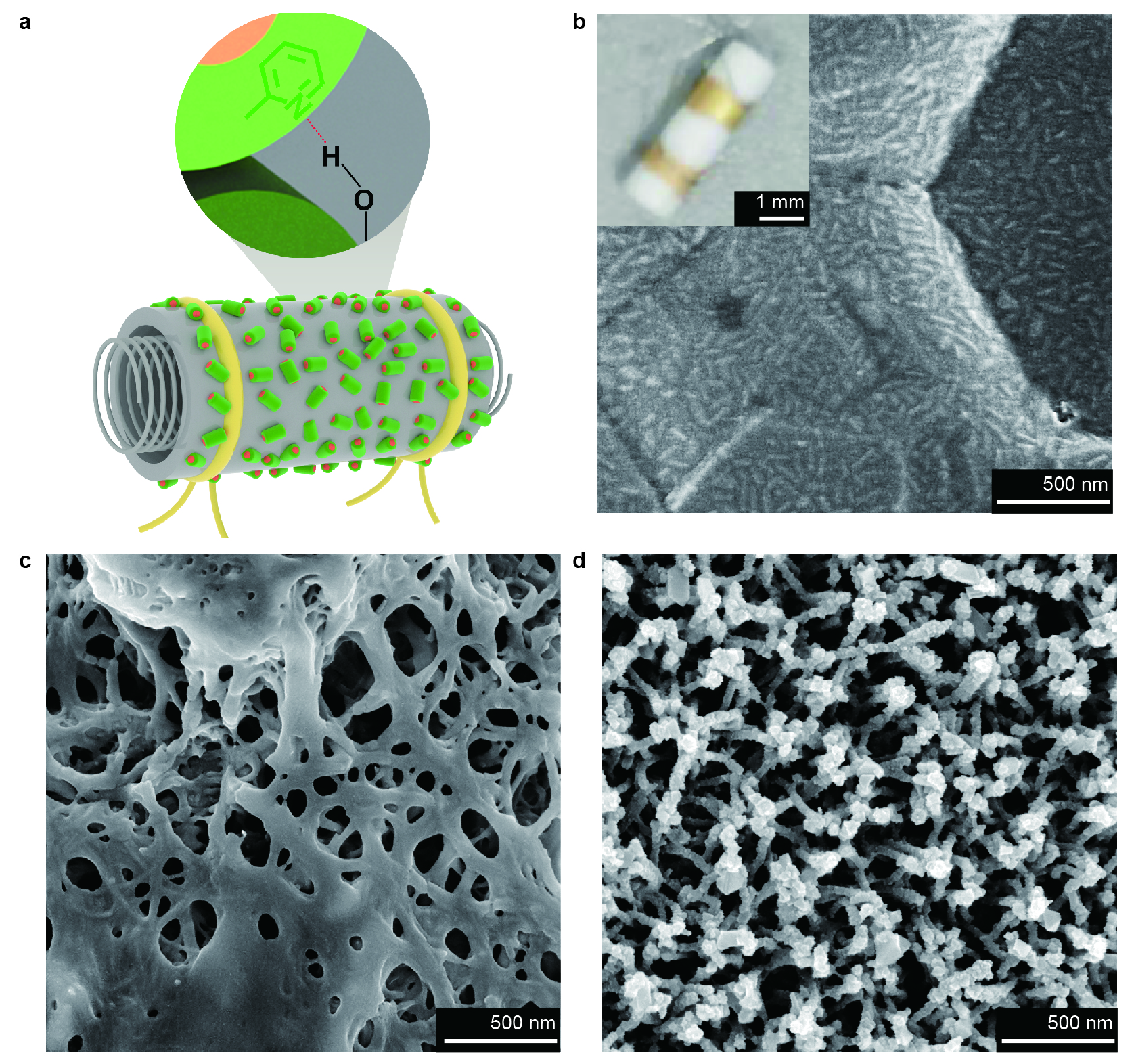


**Supplementary Figure 24. Growth of HKUST-1 nanoarray on ceramic tube. a)** Schematic illustration of the interaction between the micelle seeds and the ceramic tube. **b)** SEM image of PFS_44_-*b*-P2VP_526_ seed-coated on the ceramic tube (for seeds, *L*_n_ = 63 nm, *L*_w_ =68 nm, *L*_w_/*L*_n_ = 1.08, 0.5 mg/mL in isopropanol). Inset is a photograph of the ceramic tube loaded with the micelle seeds. **c)** SEM image of the soft nanobrush fabricated by adding 16 μL of a solution of PFS_24_-*b*-P2VP_314_ unimers (10 mg/mL in THF). **d)** SEM image of the HKUST-1 nanoarray prepared by alternatively immersing the soft nanobrush-coated ceramic tube in ethanol solutions of Cu(OAC)_2_ and H_3_BTC five times.


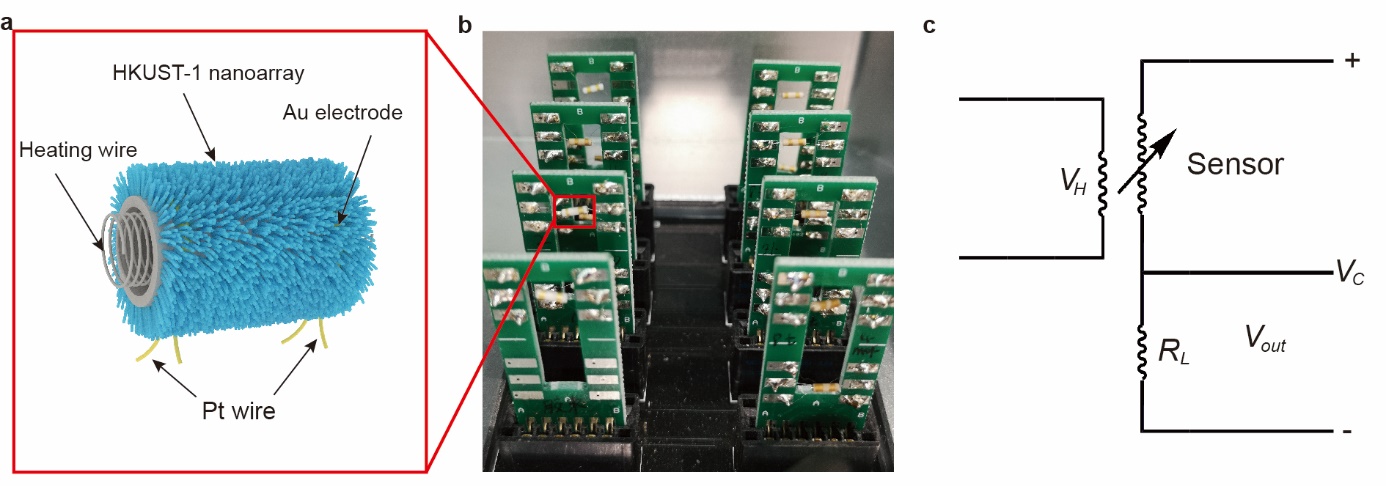


**Supplementary Figure 25. Illustration of HKUST-1 nanoarray-based gas sensor. a)** Sketch of the side-heated HKUST-1 nanoarray-based gas sensor. **b)** Photograph of the designed sensor device. **c)** Electric circuit of gas sensing measurements.


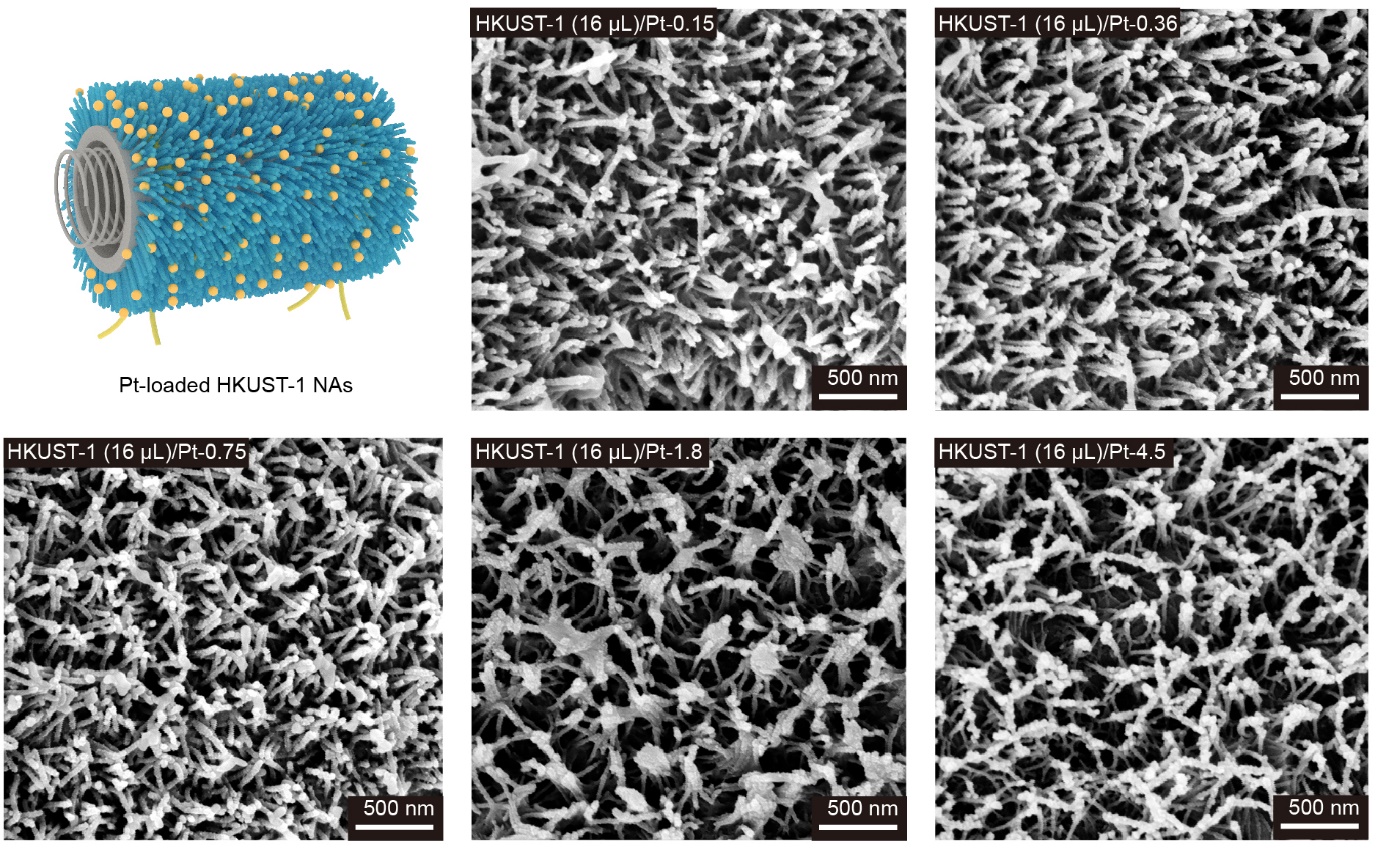


**Supplementary Figure 26. Morphology of HKUST-1 nanoarrays loaded with Pt nanoparticles.** Sketch of the gas sensor based on the HKUST-1 nanoarrays loaded with Pt nanoparticles and SEM images of the HKUST-1 nanoarrays loaded with 0.15 wt%, 0.36 wt%, 0.75 wt%, 1.8 wt% and 4.5 wt% Pt nanoparticles.


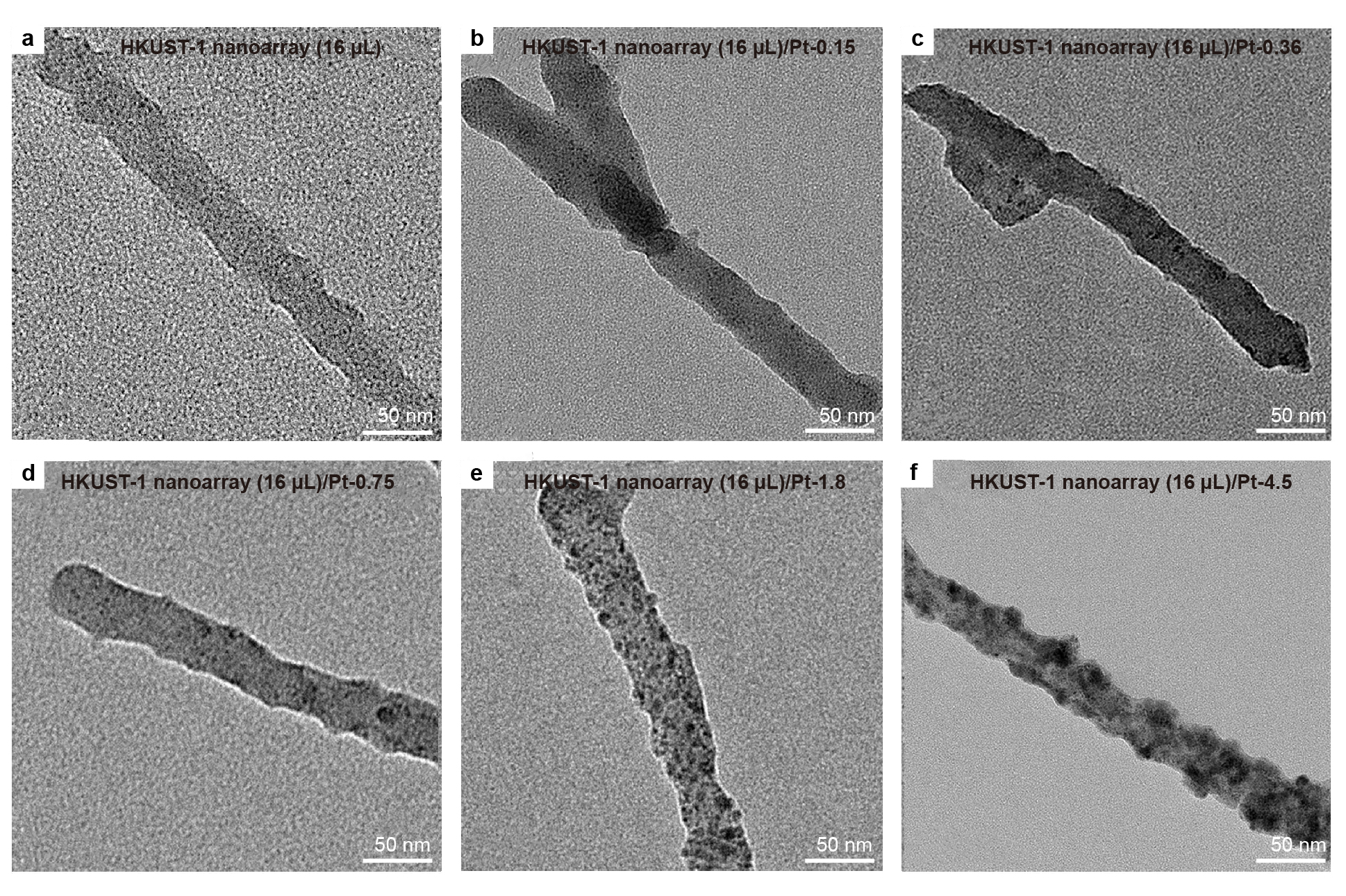


**Supplementary Figure 27. Structure of HKUST-1 nanoarrays loaded with Pt nanoparticles.** TEM images of pristine HKUST-1 nanoarray **a)**, and HKUST-1 nanoarrays loaded with 0.15 wt% **b)**, 0.15 wt% **c)**, 0.75 wt% **d)**, 1.8 wt% **e)**, and 4.5 wt% **f)** of Pt nanoparticles.


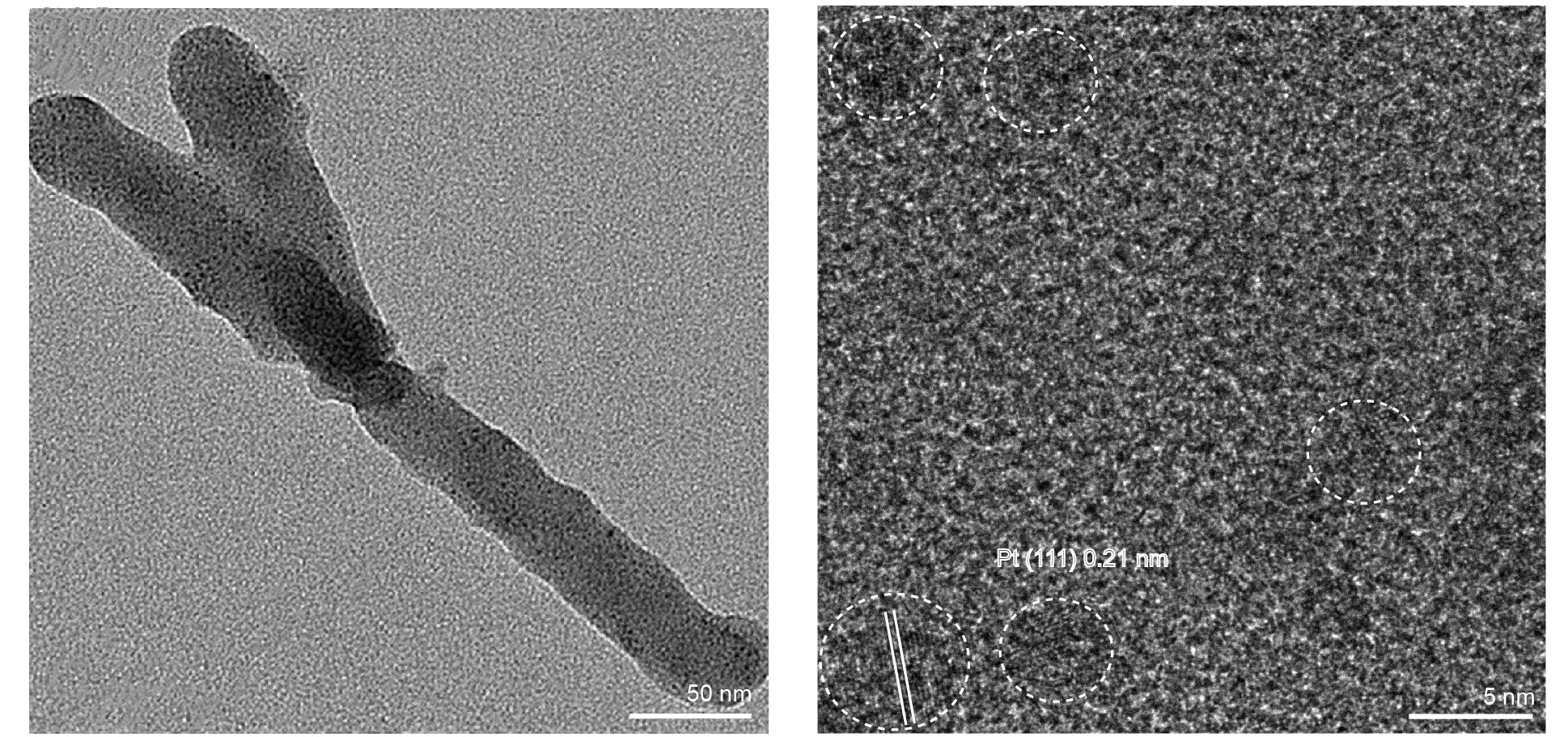


**Supplementary Figure 28.** TEM and HRTEM images of the HKUST-1 nanoarray (16 µL)/Pt-0.36.


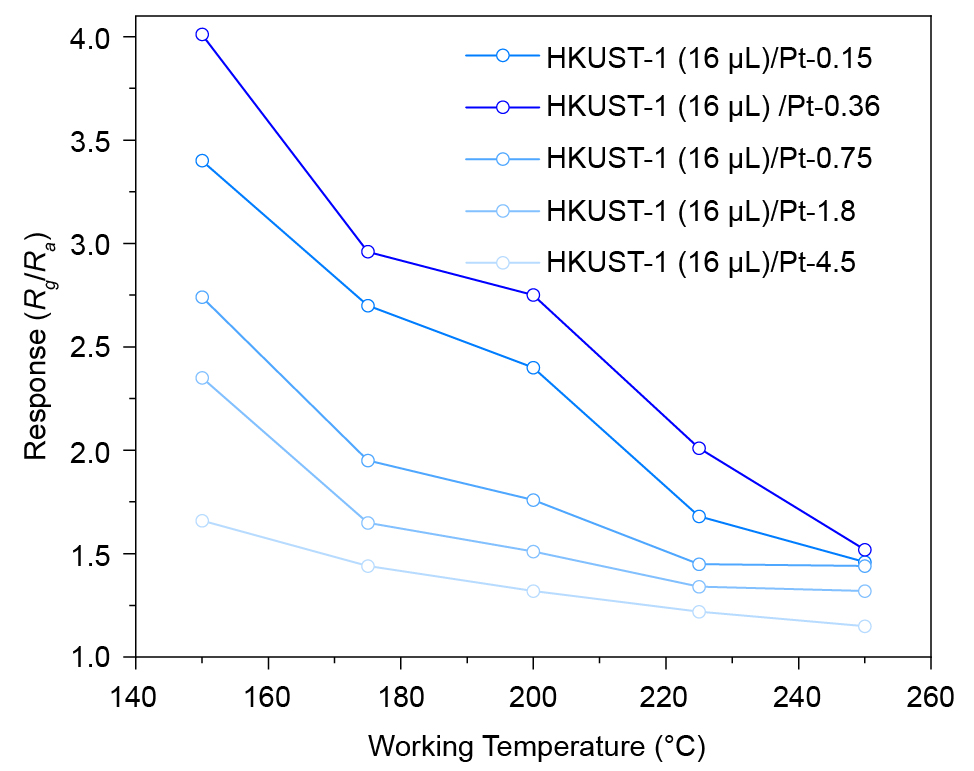


**Supplementary Figure 29.** Response of the HKUST-1 nanoarrays (16 µL)/Pt-n (n = 0.15, 0.36, 0.75, 1.8 and 4.5 wt%) to 1 ppm H_2_S at different temperatures.


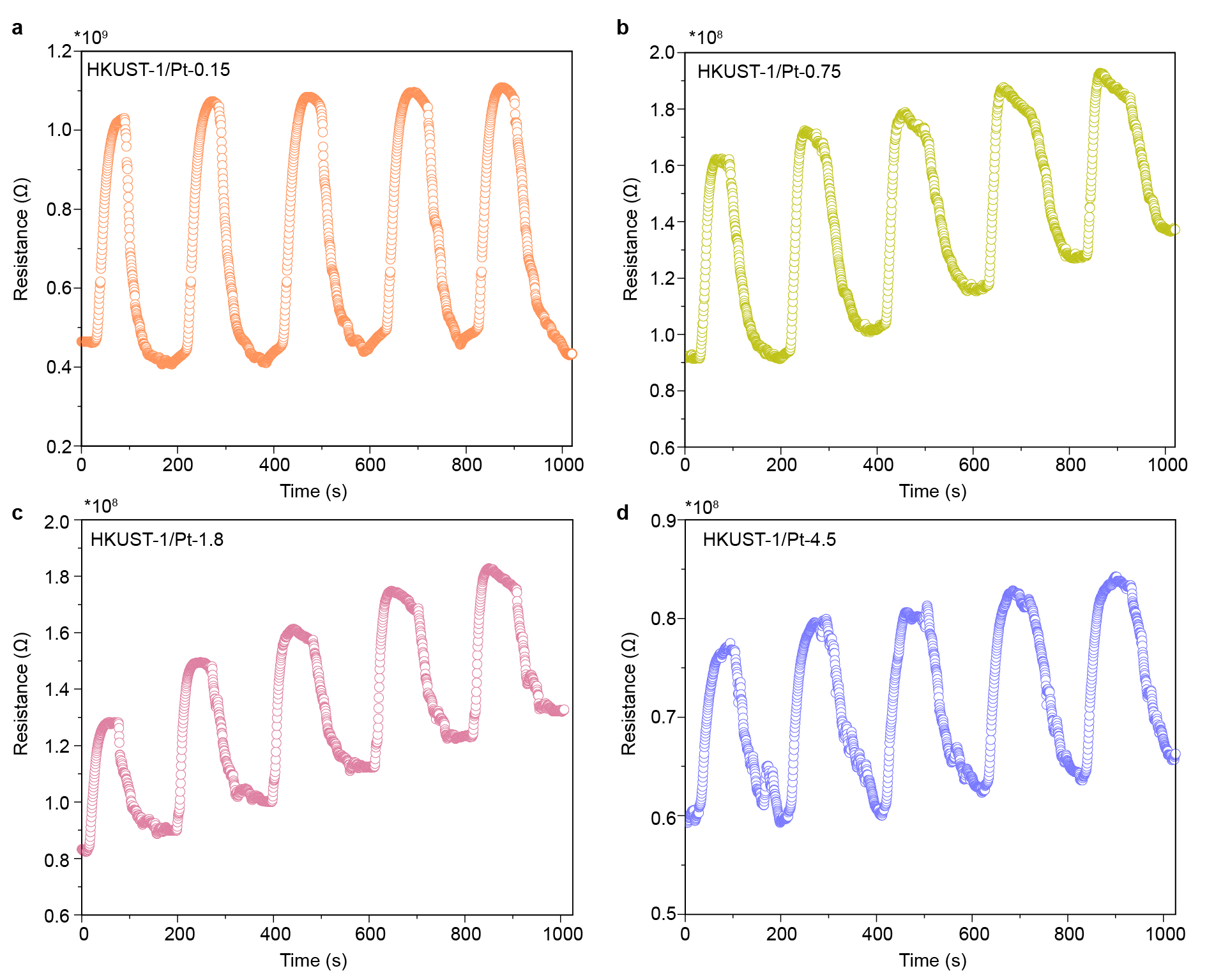


**Supplementary Figure 30. Response-recovery behavior of HKUST-1 nanoarrays (16 μL )/Pt-n** **(n = 0.15, 0.75, 1.8, 4.5 wt%) towards 1 ppm H_2_S** **at 200 °C.**


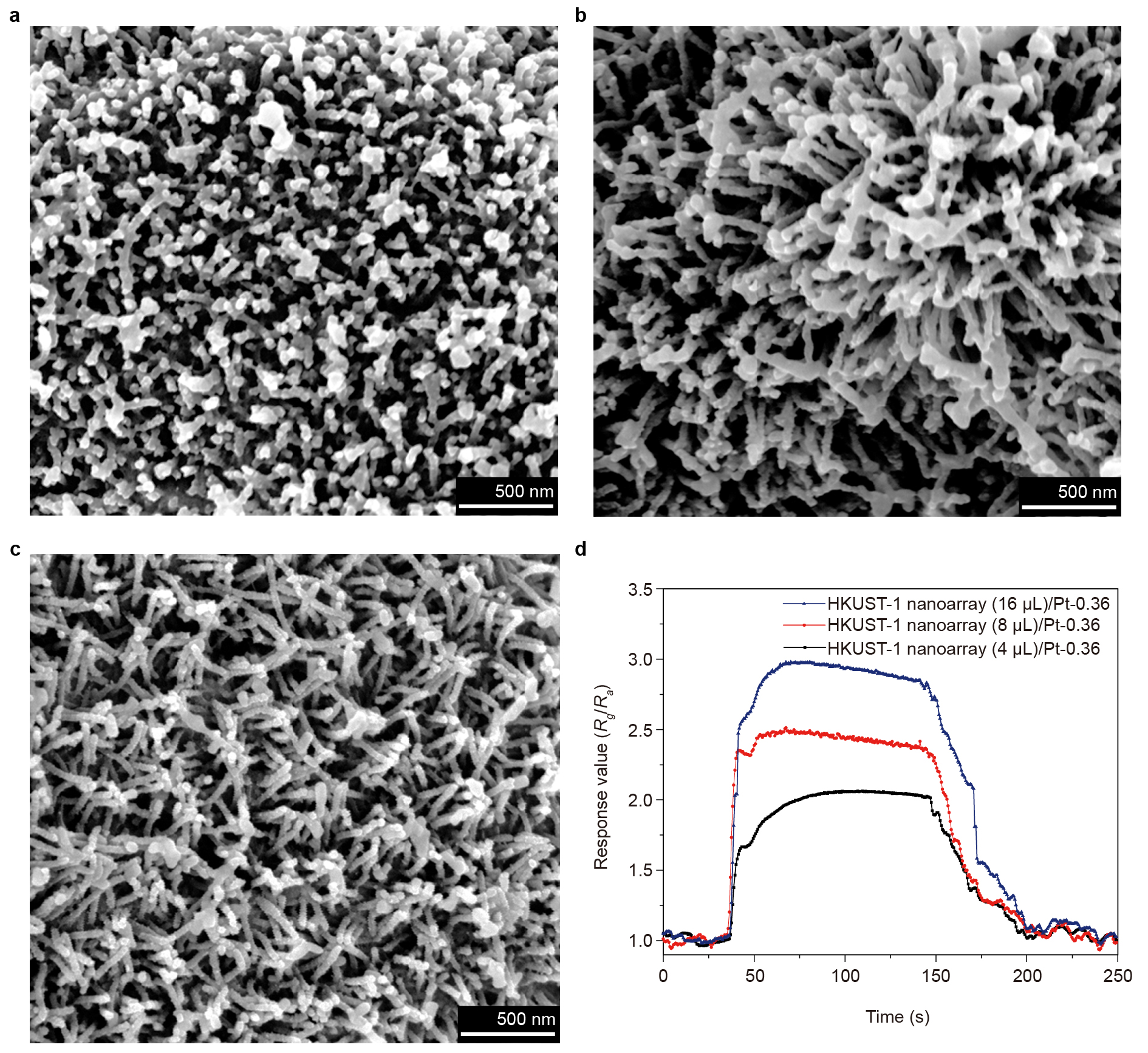


**Supplementary Figure 31. Dependence of H_2_S response on the height of HKUST-1 nanoarray. (a–c)** SEM images of the HKUST-1 nanoarrays of different lengths loaded with 0.36 wt% Pt nanoparticles, which were templated by the soft nanobrushes formed by adding 4 µL **a)**, 8 µL **b)**, and 16 µL **c)** of a solution of PFS_24_-*b*-P2VP_314_ unimers (10 mg/mL in THF), respectively. **d)** Dynamic sensing transient curves of the HKUST-1 nanoarrays/Pt-0.36 toward 1 ppm H_2_S.


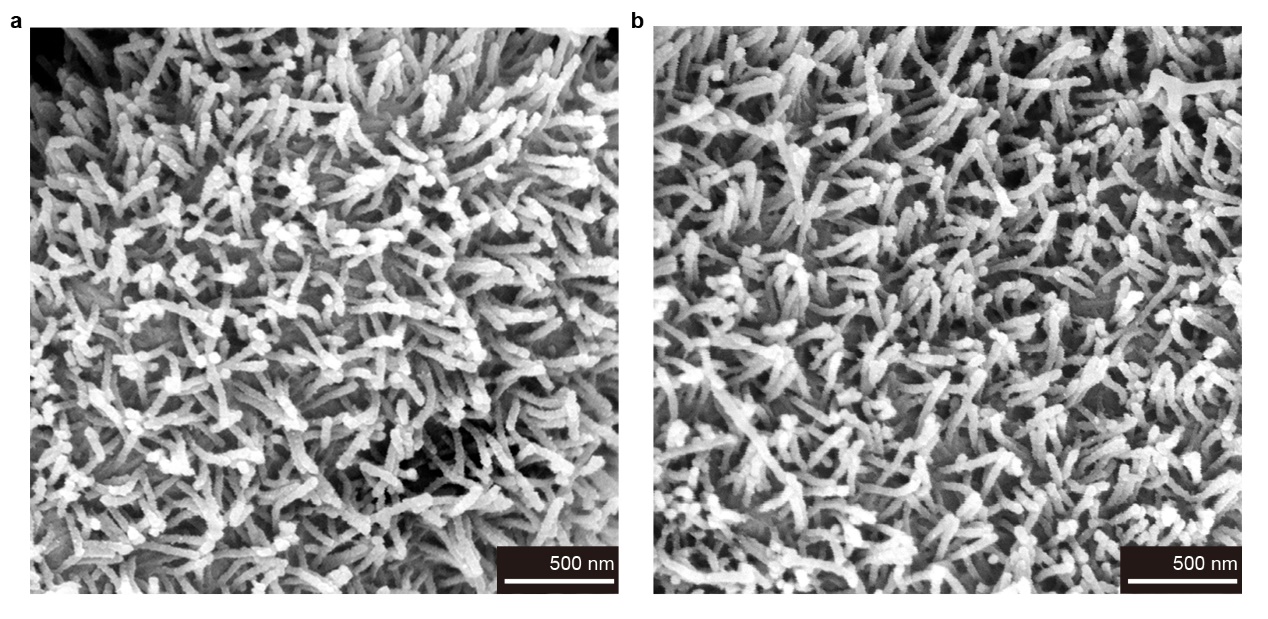


**Supplementary Figure 32. Stability of HKUST-1 nanoarray (16 µL)/Pt-0.36 over H_2_S sensing.** SEM images of the HKUST-1 nanoarray (16 µL)/Pt-0.36 **a)** before and **b)** after the catalytic reaction.


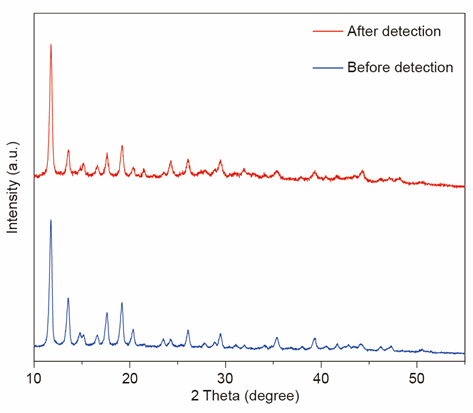


**Supplementary Figure 33. PXRD pattern of** **the HKUST-1/Pt-0.36 powder before and after the detection of 1 ppm H_2_S.**


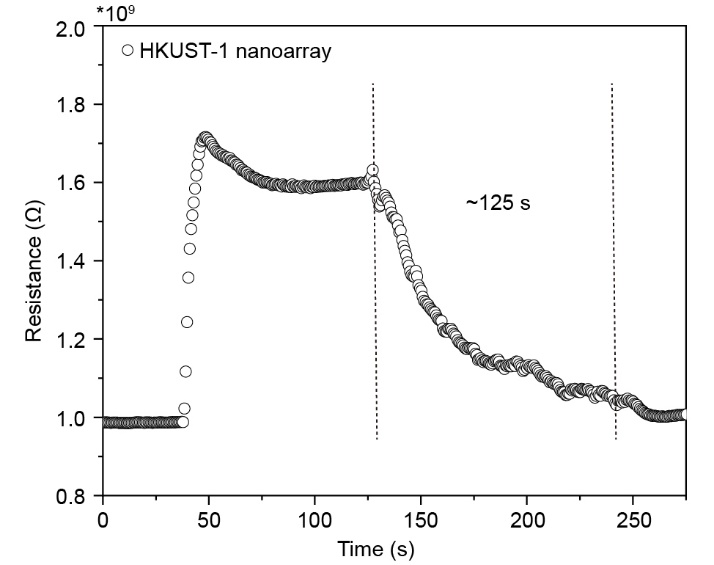


**Supplementary Figure 34.** Response–recovery curve of the pristine HKUST-1 nanoarrays (16 μL) to 1 ppm of H_2_S at 200 °C

**Supplementary Table 3.** Comparison of the H_2_S sensing performance of the reported Cu-based sensors and the HKUST-1nanoarray (16 µL)/Pt-0.36

| Sensing materials | Concentration (ppm) | Response | Temp. (°C) | Calculated LoD | Reference |
| --- | --- | --- | --- | --- | --- |
| Cu-doped SnO_2_ | 10 | 2.6 | 180 | N/A | 19 |
| Cu-In_2_O_3_ | 10 | 15.1 | 250 | N/A | 20 |
| CuO nanocuboids | 10 | 2.3 | 200 | N/A | 21 |
| CuO NPs | 10 | 9.8 | 80 | N/A | 22 |
| CuO/MoS_2_ | 10 | ~0.2 | 25 | N/A | 23 |
| CuO thin film | 5 | 3.5 | RT | N/A | 24 |
| Cu-doped ZnO | 10 | 0.75 | 250 | N/A | 25 |
| ZnO/CuO thin film | 5 | 3.5 | 250 | N/A | 26 |
| SnO_2_-CuWO_4_ | 10 | 8.64 | 335 | N/A | 27 |
| Cu_3_(HHTP)_2_ | 100 | 1 | 23 | 1000 ppb | 28 |
| CuO NPs | 5 | ~4.9 | 40 | NA | 29 |
| CuO_x_-ppy@GO | 50 | ~32.5 | RT | 43 ppb | 30 |
| HKUST-1 nanoarray (16 µL)/Pt-0.36 | 10 | 7.65 | 200 | 5 ppb | **This work** |

N/A: not applicable. The gas response of the sensor in this study is defined as *S = Ra/Rg.*


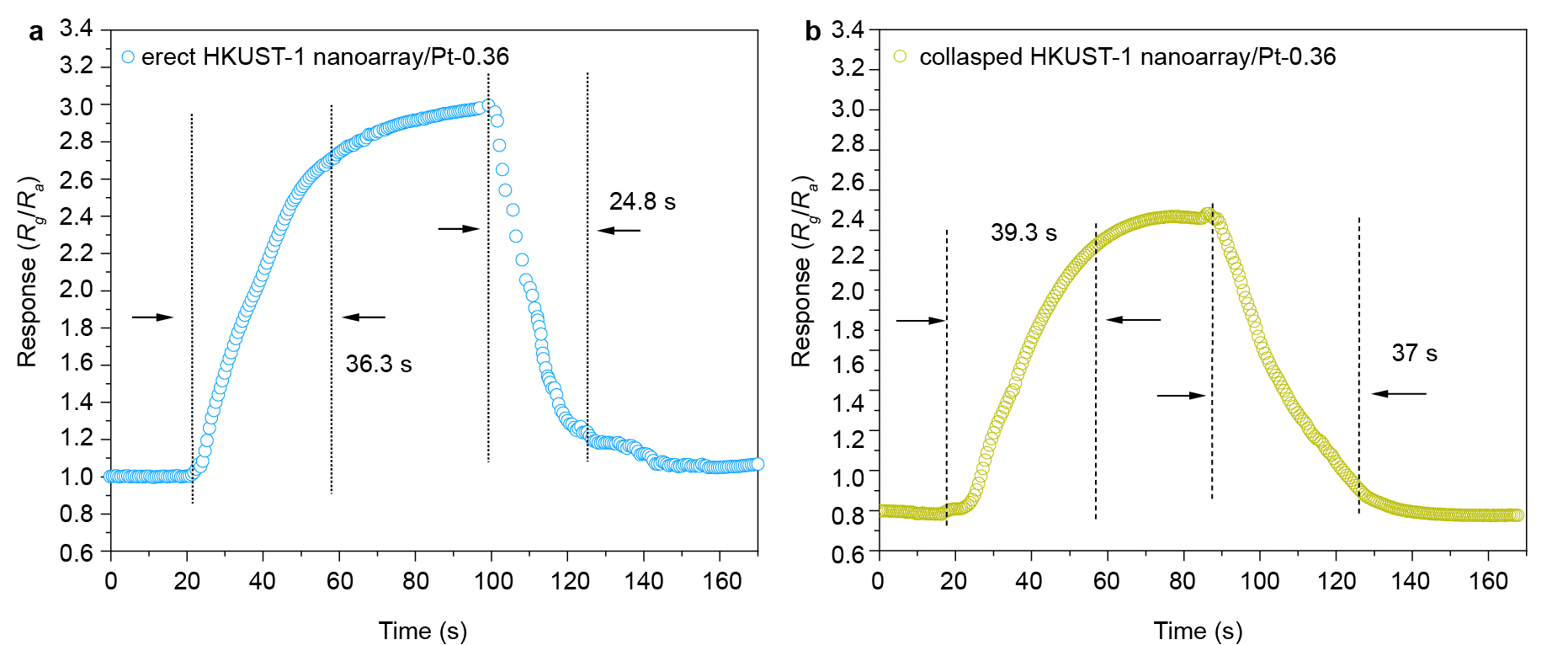


**Supplementary Figure 35. Effect of morphology on sensing performance for HKUST-1 nanoarray (16 µL)/Pt-0.36.** Response-recovery cycles of **a)** erect and **b)** collapsed HKUST-1 nanoarray (16 µL)/Pt-0.36 to 1 ppm H_2_S at 200 °C.

**Effect of Pt nanoparticles on H_2_S desorption**

Regarding the role of Pt nanoparticles in the sensing of H_2_S, we analyzed the adsorption and desorption behavior of H_2_S on the surface of HKUST-1 powder with XPS before and after loading Pt nanoparticles. After injecting H_2_S, except for the expected S 2*p* signal at 161.6 eV (Supplementary Fig. 36a), the Cu 2*p* signal of the pristine HKUST-1 powder showed two distinct new peaks at 932.8 and 952.4 eV, which can be attributed to the Cu-S bond (Supplementary Fig. 36b). The peak corresponding to the Cu-S bond retained after the desorption of H_2_S, implying a presence of strong coordination interaction between H_2_S and the Cu centers on the HKUST-1 powder. After loading the Pt nanoparticles, the Cu-S bond and the S 2*p* signal of the HKUST-1 powder/Pt-0.36 almost disappeared after the desorption of H_2_S. This was probably because the addition of Pt weakens the Cu-S bond. It was noteworthy that the peak intensity corresponding to Pt 4*f* significantly decreased after the desorption of H_2_S, while the intensity of Pt 4*f* increased (Supplementary Fig. 36c), indicating an interaction between the Pt nanoparticles and H_2_S. Given the catalytic nature of Pt nanoparticles, we speculated that the Pt nanoparticles could act as a catalyst to promote the desorption of H_2_S from HKUST-1.


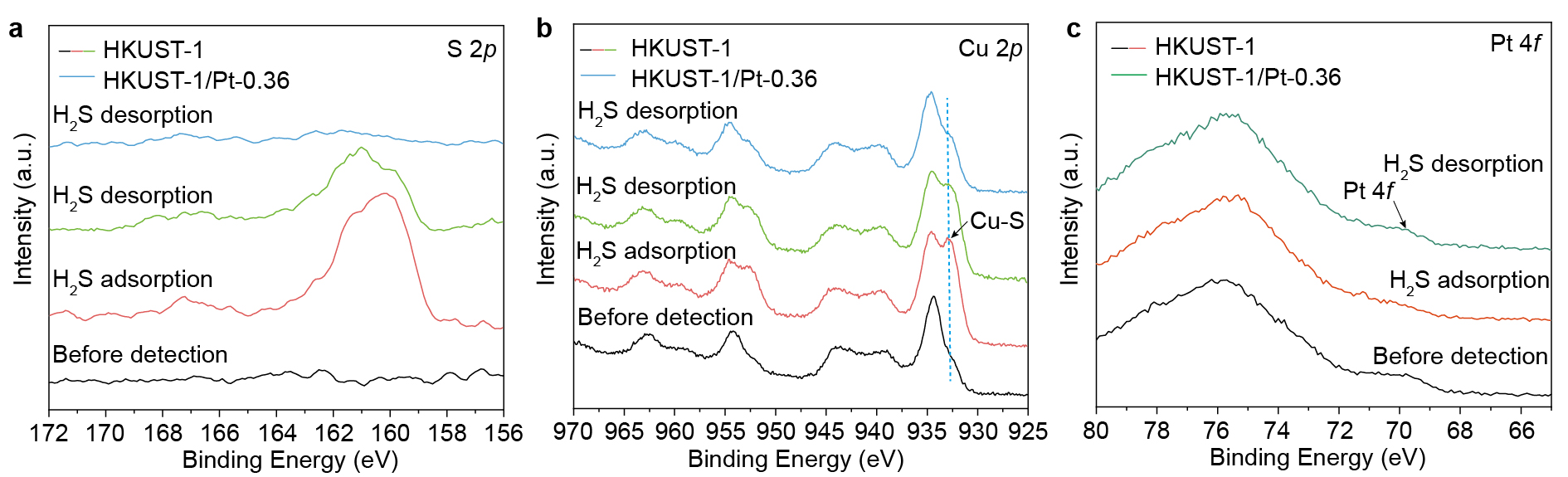


**Supplementary Figure 36. Exploration of the role of Pt in H_2_S sensing.** XPS spectra of **a)** S 2*p*, **b)** Cu 2*p*, **c)** Pt 4*f* and for the HKUST-1 powder and HKUST-1 powder/Pt-0.36.

Density functional theory (DFT) calculations were further conducted to understand the detailed mechanism.

The Vienna Ab Initio Package (VASP)^31,32^ was employed to perform all the DFT calculations within the generalized gradient approximation (GGA) using the PBE formulation.^33^ The projected augmented wave (PAW) potentials^34, 35^ were chosen to describe the ionic cores and the valence electrons were taken into account using a plane wave basis set with a kinetic energy cutoff of 400 eV. The use of the Gaussian smearing method and a width of 0.05 eV allowed for partial occupancies of the Kohn−Sham orbitals. The electronic energy was considered to be self-consistent when the energy change was smaller than 10^−5^ eV. A geometry optimization was considered as convergent when the force change was smaller than 0.02 eV/Å. Grimme’s DFT-D3 methodology^36^ was used to describe the dispersion interactions.

The equilibrium lattice constant of HKUST-1 unit cell was optimized to be *a* = 26.075 Å. It was used to construct a HKUST-1(001) surface model (model 1) with *p*(1×1) periodicity in the x and y directions and one stoichiometric layer in the z direction separated by a vacuum layer in the depth of 15 Å in order to separate the surface slab from its periodic duplicates. Model comprises of 96 H, 216 C, 144 O and 24 Cu atoms. Model 2 (HKUST-1 surface-loaded Pt clusters) was built by adding a Pt_13_ cluster onto model 1. During structural optimizations, the *Γ* point grid in the Brillouin zone was used for k-point sampling, and all atoms were allowed to relax.

The adsorption energy (*E*_ads_) of adsorbate A was defined as:

*E*_ads_ = *E*_A/surf_ - *E*_surf_ - *E*_A(g)_

where *E*_A/surf_, *E*_surf_ and *E*_A(g)_ are the energy of adsorbate A adsorbed on the surface, the energy of clean surface, and the energy of isolated A molecule in a cubic periodic box with a side length of 20 Å and a 1×1×1 Monkhorst-Pack k-point grid for Brillouin zone sampling, respectively.

The configuration of the HKUST-1/Pt nanoarray was optimized to serve as a dominant interface that interacted with H_2_S molecules (Supplementary Fig. 37a). The HKUST-1/Pt nanoarray revealed considerably higher adsorption energy (-1.41 eV) for H_2_S than that of the pristine HKUST-1 (-0.42 eV) (Supplementary Fig. 38), indicating that the Pt functionalization was beneficial for the adsorption of H_2_S. Charge density distribution was calculated to elucidate the electronic transfer behaviors during the sensing process (Supplementary Fig. 37b,c). The electron density of H_2_S near the Pt cluster in the HKUST-1/Pt nanoarray was significantly decreased compared to that of the HKUST-1 nanoarray, indicating a charge transfer from H_2_S to the Pt cluster. Bader charge analysis further revealed that the charge transfer from H_2_S to the HKUST-1/Pt nanoarray (Δ*q* = 0.27 e) was significantly higher than that to the pristine HKUST-1 nanoarray (Δ*q* = 0.11 e). These results were consistent with the experimental data and confirmed that the HKUST-1/Pt nanoarray possessed a high selectivity and sensitivity toward H_2_S.

In addition, the reaction pathway of H_2_S was evaluated to acquire a deeper understanding of the sensing process (Supplementary Fig. 37d-f). The reaction began from the co-adsorption of H_2_S and O_2_ molecule on the sensor surfaces. It should be noted that the O_2_ molecule could be activated to form atomic O at the Pt sites and displayed high affinity towards H atom. The H_2_S molecule subsequently approached the adsorbed oxygen and dissociated into adsorbed *SH. This step was thermodynamically favored by a release of 7.22 eV. As compared, the adsorbed H_2_S molecules on the pristine HKUST-1 had to overcome a higher energy barrier (1.22 eV) to obtain the intermediate product *SH. In the next step, the adsorbed SH on the HKUST-1/Pt nanoarray dissociated into adsorbed S and further converted into SO_2_ in the presence of atomic O. These results consistently demonstrated that the transformation of H_2_S molecules is dynamically and thermodynamically more favorable on the HKUST-1/Pt nanoarray.


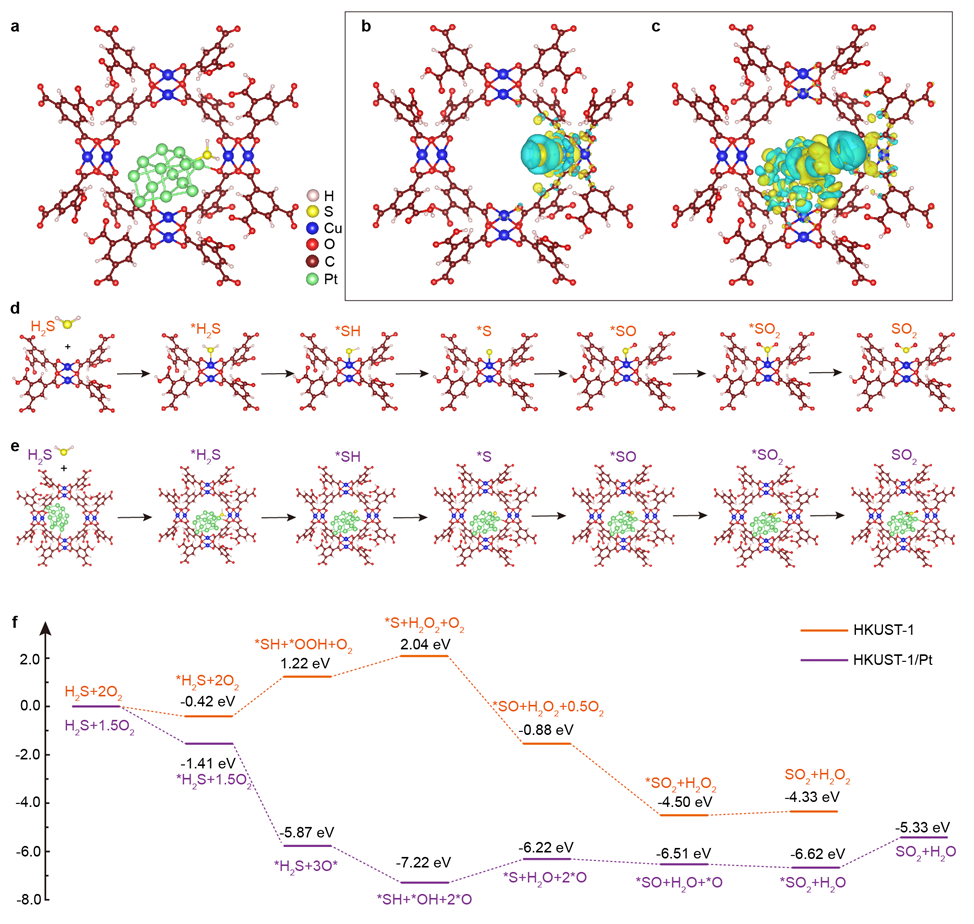


**Supplementary Figure 37. Theoretic analysis of H_2_S sensing. (a)** Optimized binding configuration of HKUST-1/Pt. **(b, c)** Charge density distributions of the HKUST-1 (b) and HKUST-1/Pt (c) nanoarrays. Yellow and light blue contours represent electron accumulation and depletion areas, respectively. **(d, e)** Geometries of intermediates involved in H_2_S oxidation on HKUST-1 (d) and HKUST-1/Pt (e) nanoarrays. (f) Relative energy diagram for the sensing of H_2_S by the HKUST-1 and HKUST-1/Pt nanoarrays.


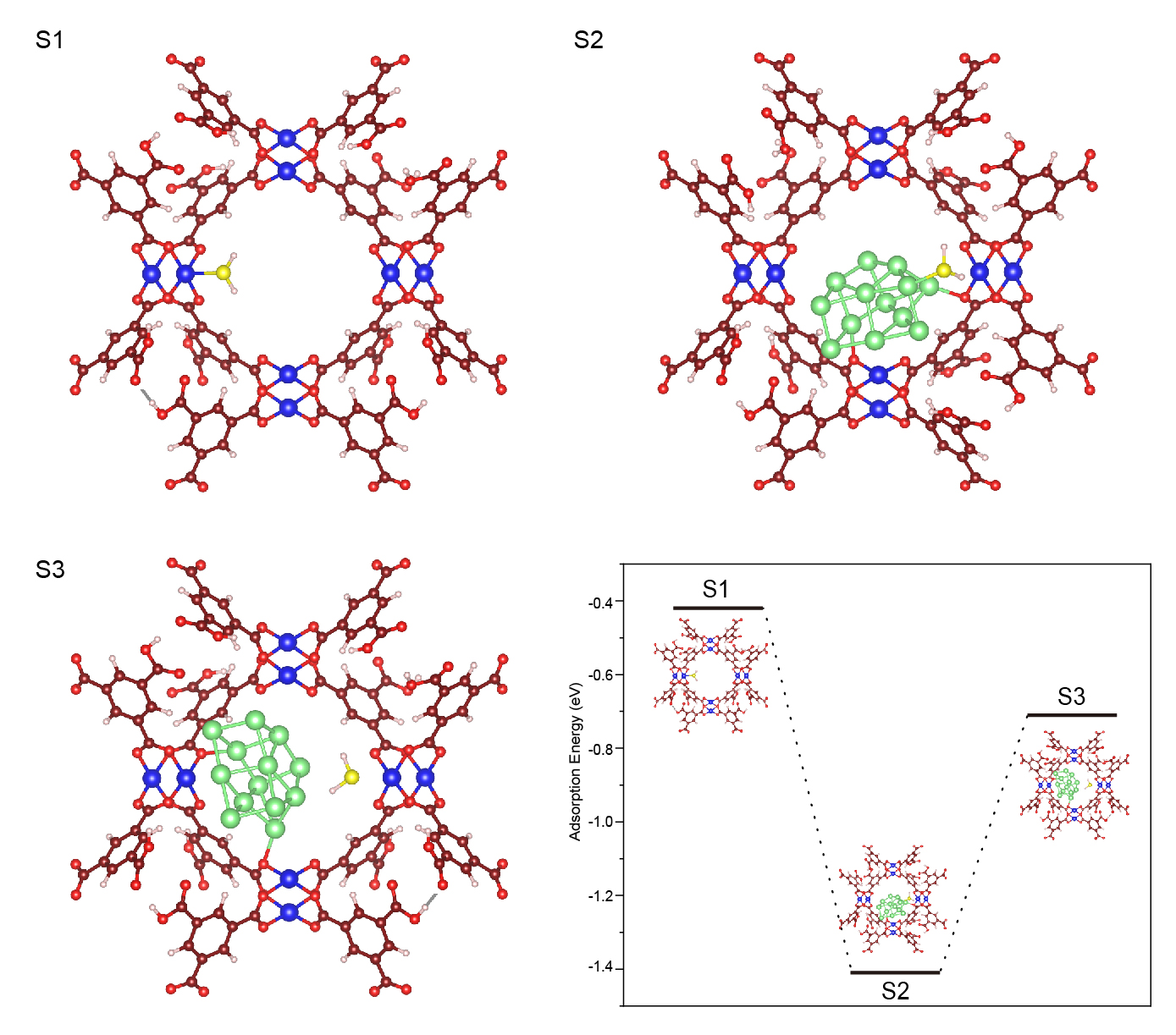


**Supplementary Figure 38.** Adsorption energy of the pristine HKUST-1 (S1), HKUST-1 sites (S3) and Pt sites (3) in HKUST-1/Pt (S2) for H_2_S.


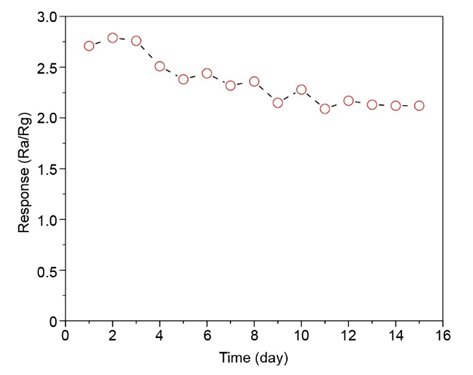


**Supplementary Figure 39.** **Sensing performance of HKUST-1 nanoarray (16µL)/Pt-0.36 towards 1 ppm H_2_S at 200 °C over two weeks.**

**Supplementary References**

1. Tsigdinos, G. A., and C. J. Hallada. Molybdovanadophosphoric acids and their salts. I. Investigation of methods of preparation and characterization. *Inorg. Chem.* **7**, 437-441 (1968).
2. Hailes, R. L. N., Oliver, A. M., Gwyther, J., Whittell, G. R. & Manners, I. Polyferrocenylsilanes: synthesis, properties, and applications. *Chem. Soc. Rev.* **45**, 5358–5407 (2016).
3. Wang, H. et al. Synthesis and self-Assembly of Poly(ferrocenyldimethylsilane-b-2-vinylpyridine) diblock copolymers. *Macromolecules* **40**, 3784–3789 (2007).
4. Brookes, C. et al. Molybdenum oxide on Fe_2_O_3_ core–shell catalysts: probing the nature of the structural motifs responsible for methanol oxidation catalysis. *ACS. Catal.* **4**, 243-250 (2013).
5. Hellier, P. et al. VO_x_/Fe_2_O_3_ shell-core catalysts for the selective oxidation of methanol to formaldehyde. *Top Catal.* **61**, 357-364 (2018).
6. Bowker, M. et al. Evolution of active catalysts for the selective oxidative dehydrogenation of methanol on Fe_2_O_3_ surface doped with Mo oxide. *Phys. Chem. Chem. Phys.* **15**, 12056-12067 (2013).
7. Bowker, M. et al. Al-doped Fe_2_O_3_ as a support for molybdenum oxide methanol oxidation catalysts. *Phys. Chem. Chem. Phys.* **22,** 18911-18918 (2020).
8. Haggblad, R. et al. Oxidation of methanol to formaldehyde over a series of Fe_1−x_Al_x_-V-oxide catalysts. *J. Catal.* **258**, 345-355 (2008).
9. Thrane, J. et al. Hydroxyapatite supported molybdenum oxide catalyst for selective oxidation of methanol to formaldehyde: studies of industrial sized catalyst pellets. *Catal. Sci. Technol.* **11**, 970-983 (2021).
10. Massa, M. et al. Oxidation of methanol to formaldehyde on cation vacant Fe–V–Mo-oxide. *Appl. Catal. A: Gen.* **408**, 63-72 (2011).
11. Shimoda, K. et al. Synthesis of high dimensionally structured Mo-Fe mixed metal oxide and its catalytic activity for selective oxidation of methanol. *Inorg. Chem.* **59**, 5252-5255 (2020).
12. Jin, G. et al. Fe_2_(MoO_4_)_3_/MoO_3_ nano-structured catalysts for the oxidation of methanol to formaldehyde. *J. Catal.* **296**, 55-64 (2012).
13. Liu, H. and E. Iglesia. Selective one-step synthesis of dimethoxymethane via methanol or dimethyl ether oxidation on H_3+n_VnMo_12-n_PO_40_ Keggin structures. *J. Phys. Chem. B.* **107**, 10840-10847 (2003).
14. I. E. Wachs and R. J. Madix. The oxidation of methanol on a silver (110) catalyst. *Surf. Sci.* **76**, 531-558 (1978).
15. C. Louis et al. Catalytic properties of silica-supported molybdenum catalysts in methanol oxidation: the influence of molybdenum dispersion. *J. Catal.* **109**, 354-366 (1988).
16. Xie, Y. et al. Investigation of the reactions of small neutral iron oxide clusters with methanol. *J Chem. Phys.* **130**, 114306-114318 (2009).
17. Bruckman, K. et al. Catalytic behavior of unsupported and heteropolysalt-supported H_3+n_PMo_12–n_V_n_O_40_ heteropolyacids in the test reaction of CH_3_OH oxidation. *J. Catal*. **139**, 455-467 (1993).
18. J. G. Highfield. Elucidation of the mechanism of dehydration of methanol over 12-tungstophosphoric acid using infrared photoacoustic spectroscopy. *J. Catal.* **95**, 108-119 (1985).
19. Zhang, S. et al. Facile fabrication of a well-ordered porous Cu-doped SnO_2_ thin film for H_2_S sensing. *ACS Appl. Mater. Interfaces.* **6**, 14975-14980 (2014).
20. Zhang, Y. et al. Electrospun Cu-doped In_2_O_3_ hollow nanofibers with enhanced H_2_S gas sensing performance. *J. Adv. Ceram.* **11**, 427-442 (2022).
21. Dhakshinamoorthy, J., and B. Pullithadathil. New insights towards electron transport mechanism of highly efficient p-Type CuO (111) nanocuboids-based H_2_S gas sensor. *J. Phys. Chem. C.* **120**, 4087-4096 (2016).
22. Ayesh, A. I. et al. Selective H_2_S sensor based on CuO nanoparticles embedded in organic membranes. *Sens. Actuators B.* **231**, 593-600 (2016).
23. Zhang, D., Wu, J., and Cao, Y. Ultrasensitive H_2_S gas detection at room temperature based on copper oxide/molybdenum disulfide nanocomposite with synergistic effect. *Sens. Actuators B.* **287**, 346-355 (2019).
24. Ramgir, N. S. et al. Sub-ppm H_2_S sensing at room temperature using CuO thin films. *Sensors and Actuators B.* **151,** 90-96 (2010).
25. Girija, K. G. et al. Highly selective H_2_S gas sensor based on Cu-doped ZnO nanocrystalline films deposited by RF magnetron sputtering of powder target. *J. Alloys Compd.* **684**, 15-20 (2016).
26. Han, C. et al. Composition-controllable p-CuO/n-ZnO hollow nanofibers for high-performance H2S detection. *Sens. Actuators B.* **285**, 495-503 (2019).
27. Simion, C. E. et al. H_2_S sensing mechanism of SnO_2_-CuWO_4_ operated under pulsed temperature modulation. *Sens. Actuators B.* **259**, 258-268 (2018).
28. Ali, A. et al. Flexible Cu_3_(HHTP)_2_ MOF membranes for gas sensing application at room temperature. *Nanomaterials.* **12**, 913 (2022).
29. Peng, F. et al. Studies on sensing properties and mechanism of CuO nanoparticles to H_2_S gas. *Nanomaterials.* **10,** 744 (2020).
30. Song, Z. et al. Sensitive room-temperature H_2_S gas sensors employing SnO_2_ quantum wire/reduced graphene oxide nanocomposites. *Chem. Mater.* **28**, 1205-1212 (2016).
31. Kresse, G.; Furthmüller, J. Efficiency of Ab-Initio Total Energy Calculations for Metals and Semiconductors Using a Plane-Wave Basis Set. Comput. Mater. Sci. 6, 15-50 (1996).
32. Kresse, G.; Furthmüller, J. Efficient Iterative Schemes for Ab Initio Total-Energy Calculations Using a Plane-Wave Basis Set. Phys. Rev. B 54, 11169−11186 (1996).
33. Perdew, J. P.; Burke, K.; Ernzerhof, M. Generalized Gradient Approximation Made Simple. Phys. Rev. Lett. 77, 3865−3868 (1996).
34. Kresse, G.; Joubert, D. From Ultrasoft Pseudopotentials to the Projector Augmented-Wave Method. Phys. Rev. B 59, 1758-1775 (1996).
35. Blöchl, P. E. Projector Augmented-Wave Method. Phys. Rev. B 50, 17953−17979 (1994).
36. Grimme, S.; Antony, J.; Ehrlich, S.; Krieg, H. J. Chem. Phys. 132, 154104 (2010).
